# Analysis of Temperature-Programmed Desorption via Equilibrium Thermodynamics

**DOI:** 10.1021/acsphyschemau.2c00031

**Published:** 2022-11-15

**Authors:** Michael Schmid, Gareth S. Parkinson, Ulrike Diebold

**Affiliations:** Institute of Applied Physics, TU Wien, 1040Vienna, Austria

**Keywords:** temperature-programmed desorption, adsorption energy, thermodynamics, chemical potential, thermal desorption spectroscopy

## Abstract

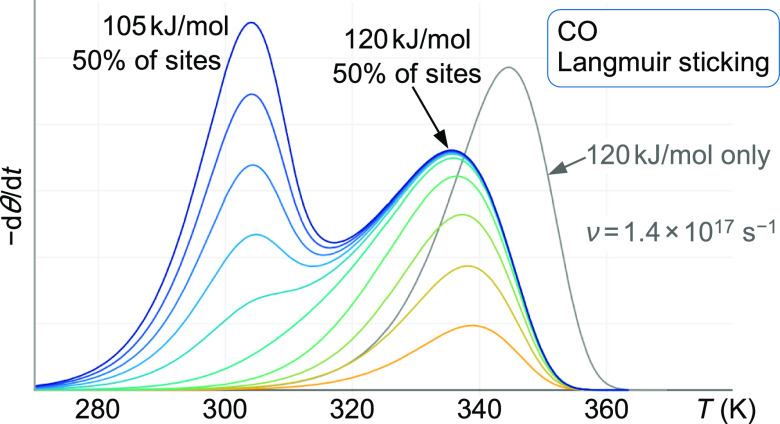

Temperature-programmed desorption (TPD) experiments in surface science are usually analyzed using the Polanyi–Wigner equation and/or transition-state theory. These methods are far from straightforward, and the determination of the pre-exponential factor is often problematic. We present a different method based on equilibrium thermodynamics, which builds on an approach previously used for TPD by Kreuzer et al. (*Surf. Sci.***1988**). Equations for the desorption rate are presented for three different types of surface–adsorbate interactions: (i) a 2D ideal hard-sphere gas with a negligible diffusion barrier, (ii) an ideal lattice gas, that is, fixed adsorption sites without interaction between the adsorbates, and (iii) a lattice gas with a distribution of (site-dependent) adsorption energies. We show that the coverage dependence of the sticking coefficient for adsorption at the desorption temperature determines whether the desorption process can be described by first- or second-order kinetics. The sticking coefficient at the desorption temperature must also be known for a quantitative determination of the adsorption energy, but it has a rather weak influence (like the pre-exponential factor in a traditional TPD analysis). Quantitative analysis is also influenced by the vibrational contributions to the energy and entropy. For the case of a single adsorption energy, we provide equations to directly convert peak temperatures into adsorption energies. These equations also provide an approximation of the desorption energy in cases that cannot be described by a fixed pre-exponential factor. For the case of a distribution of adsorption energies, the desorption spectra cannot be considered a superposition of desorption spectra corresponding to the different energies. Nevertheless, we present a method to extract the distribution of adsorption energies from TPD spectra, and we rationalize the energy resolution of TPD experiments. The analytical results are complemented by a program for simulation and analysis of TPD data.

## 1 Introduction

Temperature-programmed desorption (TPD) [also named thermal desorption spectroscopy (TDS)] is the most common experimental technique for obtaining quantitative information about adsorption energies. TPD analysis for experiments under ultrahigh vacuum, where readsorption is negligible, is usually based on the Polanyi–Wigner equation^1,2^
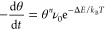
1where θ is the coverage, *n* is the desorption order, ν_0_ is the pre-exponential factor, and *T* is the temperature. Δ*E* is the desorption barrier. Assuming barrierless adsorption, this is the energy difference between the gas and the adsorbed phase. (We employ the sign convention that Δ*E* is positive for a stable adsorbate, and we take the adsorption energy or enthalpy of a stable adsorbate as a negative number.) To determine Δ*E* from experimental TPD data, that is, from the desorption rate −dθ/d*t*, the pre-exponential factor ν_0_ must be known. Assuming that ν_0_ is constant, in principle, it can be determined by varying the heating rate β = d*T*/d*t* in the TPD experiment.^3^ Unfortunately, a rather large variation of β is required (at least 1–2 orders of magnitude^1,4^), but in experiments, the range of β is quite limited. On the high-β side, the limitation is due to finite pumping speed and temperature gradients in the sample; at low β, the desorption rates are too low for accurate measurements with a mass spectrometer. Other approaches to determine ν_0_ are based on TPD curves with different initial coverages.^5,6^ All these methods become questionable when ν_0_ strongly changes; it will be shown later how the coverage dependence of the sticking coefficient affects the desorption rate (and, hence, the coverage dependence of ν_0_). Two rather simple methods are the determination of ν_0_ from the width of the desorption peak or the peak shape,^1,7,8^ but this approach becomes invalid if the peak is broadened by interaction between adsorbates or variations in adsorption energy between different sites. Another technique based on the shape of a single desorption peak is leading edge analysis,^9,10^ which employs the low-temperature side of the peaks to determine ν_0_; also, this method is not always suitable for the case of a distribution of adsorption energies. It will be shown below that these methods can also fail in the case of precursor-mediated adsorption.

The pre-exponential factor ν_0_ can also be determined from theory; this is usually based on transition-state theory (TST).^11^ A recent paper by Campbell et al. provides an overview of the underlying models for the adsorbed state, which influences the ν_0_ value.^12^ Unfortunately, the application of TST to TPD is far from straightforward, and the equations in the literature^1,12−15^ are not always consistent.

In the current work, we will take a different approach, purely based on equilibrium thermodynamics, bypassing the use of TST. This approach was already used in 1980 for adsorption isobars.^16^ For TPD, it is mainly useful in the case of non-activated adsorption and was pioneered by Kreuzer and Payne et al.^17−23^ for a lattice gas at the surface, even including a second layer.^24^ The work of Kreuzer and Payne et al. was mainly focusing on interactions between adsorbates, but the approach never gained the popularity it would deserve as a basis for TPD analysis. (The lack of widespread usage may be due to the complexity of dealing with the Ising model in the works of Kreuzer and Payne et al. The approach can also be used with simpler interaction models, however.^16,25^) Later, the same approach was suggested (but not carried out fully) in ref (12). Here, we will follow the equilibrium approach in a slightly different way, also establishing a direct link to the common TPD analysis using eq 1. For the case of an ideal lattice gas describing the adsorbate and first-order desorption, we will provide an equation for the pre-exponential factor and show that (and why) its value is often much higher than the often assumed value of *k*_B_*T*/*h* ≈ 10^13^ s^–1^. For single adsorption energies, we provide equations for direct determination of the adsorption energy from TPD peaks. We also provide a method for simulating and analyzing TPD curves for an inhomogeneous surface, with a distribution of adsorption energies varying from site to site, and we provide a computer program for simulating TPD curves and determination of adsorption energy distributions. The application of our method (and program) to a real-world example, CO/Fe_3_O_4_, is presented in the Supporting Information.

## 2 Equilibrium Thermodynamics as a Basis

In a *gedankenexperiment*, we could stop the heating ramp of a TPD experiment at any temperature *T* and apply an external gas with the same temperature as the sample and a suitable pressure *p* to obtain adsorption–desorption equilibrium.^26^ In this situation, the time between adsorption and desorption events at a given adsorption site is in the order of seconds, much longer than the timescale of individual adsorption or desorption events (<10^–12^ s); thus, adsorption and desorption events are independent of each other. Therefore, in equilibrium, the desorption rate (corresponding to the negative time derivative of the coverage θ observed in a TPD experiment) equals the product of the sticking coefficient *s* and the impingement rate of the gas molecules^19^
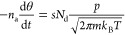
2Here, *p* is the pressure, *m* is the mass of one gas molecule, and *n*_a_ is the density of adsorption sites (per area). An adsorbate density of *n*_a_ corresponds to θ = 1. The fraction at the right-hand side is the impingement rate. The factor *N*_d_ takes dissociation of molecules into account; *N*_d_ = 1 for molecular adsorption and *N*_d_ = 2 describes a diatomic molecule dissociating into two atoms of equal kind (e.g., dissociation of H_2_; the current paper does not consider dissociation into unequal fragments such as H_2_O → H + OH).^27^ It is important that *s* is the sticking coefficient at the desorption temperature. The sticking coefficient *s* may depend on the (kinetic, vibrational, or rotational) energy of the molecules. In such a case, one has to take a weighted average of the (energy-dependent) *s* values, with weights corresponding to the fraction of molecules with the given energy impinging on the surface. Unless there is a substantial energy barrier on adsorption, we can assume an energy-independent value of *s* because *s* and the impingement rate only enter the pre-exponential factor ν_0_ (see below), and the calculated adsorption energy is not very sensitive to ν_0_.

In equilibrium, the chemical potentials μ_a_ and μ_g_ of the adsorbed phase and the gas are equal (with *N*_d_ taking into account that we define μ_a_ for the adsorbed atoms in the case of dissociation)
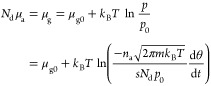
3Here, we have assumed an ideal gas. μ_g0_ is the chemical potential in the gas phase at the reference pressure *p*_0_ (10^5^ Pa). The final expression in eq 3 was obtained with the gas pressure *p* from eq 2.

Since we consider equilibrium at a constant temperature and pressure, for each of the phases, the chemical potential can be directly expressed via the Gibbs free energy
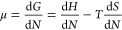
4As long as the quantities *H*/*N* and *S*/*N* (the enthalpy and entropy per molecule, respectively) do not depend on the number of molecules *N*, we can replace the derivatives in eq 4 by the respective quantity per molecule. This is the case for an ideal gas. For simplicity, in the following, we will discuss quantities per molecule (or per mole, if the Boltzmann constant is replaced by the gas constant *R*). For many gases, *H* and *S* are tabulated as a function of temperature for atmospheric pressure *p*_0_.^28,29^ This allows us to directly determine μ_g0_ for the gas phase.

For gases (or temperatures) where tabulated data are not available, the gas-phase entropy per molecule^31^ at the reference pressure *p*_0_ can be written as a sum of two contributions: that of the translational degrees of freedom, given by the Sackur–Tetrode equation, and the entropy per molecule *S*_g,int_ of the internal degrees of freedom. (Vibrations and rotations; for an ideal gas, these do not depend on pressure.)
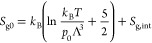
5Λ is the thermal de Broglie wavelength of the gas molecules
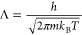
6Also, the enthalpy per gas-phase molecule has contributions from translation (the enthalpy of an ideal monatomic gas) and from the internal degrees of freedom
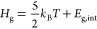
7

For vibrations, assuming a harmonic oscillator model with a frequency ν, ignoring the zero-point energy,^32^ the contributions to the energy and entropy are^12,33,34^
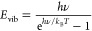
8
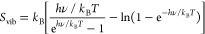
9

For rotations in the gas phase, we have to distinguish two cases. For linear molecules (two axes of molecular rotations), with the exception of hydrogen at low temperatures (where very few rotational states are occupied and quantization must be taken into account), we have^33,35^
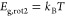
10
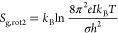
11Here, *I* is the moment of inertia and σ = 2 for molecules with mirror symmetry (N_2_, C_2_H_2_, etc.) and σ = 1 otherwise (CO, NO, HCN, etc.). For molecules with three axes of rotation, the following applies^33,35^
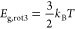
12
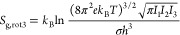
13where *I*_1_, *I*_2_, and *I*_3_ are the principal moments of inertia and σ is the number of indistinguishable rotational orientations of the molecule (e.g., σ = 12 for CH_4_; the molecule can be rotated such that a given H atom takes four different positions, and for each of these, three 120° rotations around the axis between that H atom and C result in the same configuration).^33^ It should be noted that there is no need to calculate the individual values of *I*_1_, *I*_2_, and *I*_3_; their product is the product of the eigenvalues of the inertia tensor and, thus, equal to the determinant of the inertia tensor. *S*_g,int_ and *E*_g,int_ are then obtained as sums over the contributions of each vibrational degree of freedom and the rotational contributions.

The chemical potential at the reference pressure then becomes (again using quantities per molecule)
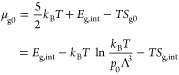
14where *E*_g,int_ and *S*_g,int_ are the sums over all vibrational contributions from eqs 8 and 9 and the appropriate rotational term (either eqs 10 and 11 or 12 and 13). Note that in eq 14, the enthalpy of translation of the gas cancels out with the 5/2 term from the Sackur–Tetrode eq 5.

Also for the adsorbate phase, the chemical potential can be split into several contributions. The pressure term in the enthalpy is negligible for the adsorbate (*pV* ≲ 10^–14^ eV per molecule in a typical TPD experiment); thus, we can write all enthalpies *H* of the adsorbate as energies *E*. Assuming that we can express all energy and entropy terms as quantities per adsorbed atom or molecule, we can write
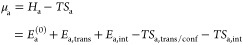
15The energy of translation *E*_a,trans_ is present only in the case of the adsorbates forming a two-dimensional (2D) gas with free translation. *S*_a,trans/conf_ is the entropy of this translation in the case of a 2D gas with free translation. For a lattice gas, *S*_a,trans/conf_ is the derivative of the configurational entropy *S*_conf_ with respect to particle number, see Section 4. Then, *S*_conf_ is related to distributing the adsorbates to the adsorption sites. For the internal degrees of freedom (*E*_a,int_ and *S*_a,int_), we have to sum up the contributions of vibrations (including hindered translation in case the adsorbate is not a 2D gas with free translation), rotation around a bond axis (if any), as well as configurational contributions to the entropy if the adsorbate has different stable configurations at a given site, for example, different azimuthal orientations of a flat-lying linear molecule on a surface with 3-fold rotational symmetry. (In our computer program, the latter contributions can be entered as *S*_extra_.) *E*_a_^(0)^ is the adsorption energy without the vibrational, rotational, and (for an ideal 2D gas) translational energy contributions; in other words, *E*_a_^(0)^ can be seen as the strength of the chemical bond between the adsorbate and the surface. When ignoring temperature-dependent changes of the substrate, this is the same as the adsorption energy in the *T* → 0 limit.^36^ Thus, the *E*_a_^(0)^ value can be directly compared with a density functional theory (DFT) calculation. One has to bear in mind that zero-point corrections should be applied to the DFT values (especially for hydrogen).

With these contributions to the chemical potentials and eq 3, the desorption rate can be expressed as
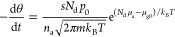
16
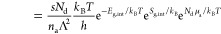
17
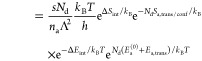
18with
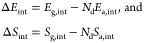
19For the case of dissociating gases (*N*_d_ = 2), we take energies and entropies per *atom* of the adsorbed phase; this explains the factors *N*_d_ in eq 19 and in the adsorbate-related exponents of eqs 16–18.

In the following sections, we will discuss various possibilities for the adsorbate: an ideal 2D hard-sphere gas and well-defined adsorption sites (i.e., a 2D lattice gas), analogous to ref (12). For the lattice gas model, we will consider the cases of a single adsorption energy and a distribution of adsorption energies.

## 3 Case 1: Adsorbates Are an Ideal (Hard-Sphere) 2D Gas

If the potential energy surface for adsorbate atoms or molecules is flat, that is, its spatial variations are small with respect to *k*_B_*T*, and adsorbate–adsorbate interactions are negligible (apart from avoiding overlap of the molecules, which are treated as hard disks), we can treat the adsorbate as an ideal 2D gas but with an excluded area *a* per molecule (in analogy to the excluded volume *b* of a van der Waals gas).^37^ For this 2D gas, the translational entropy per molecule is given by the 2D version of the Sackur–Tetrode equation^39^ (but with an excluded area)
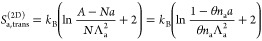
20where *N* is the number of molecules and *A* is the surface area. The 2D gas has no adsorption sites; nevertheless, for consistency with the case of a lattice gas, we express the areal density of molecules *N*/*A* as θ*n*_a_; here, *n*_a_ is the areal density of the molecules at saturation coverage (θ = 1). If the adsorbate and the gas-phase species are the same molecule, Λ_a_ = Λ. In the case of dissociation, the thermal de Broglie wavelength of the adsorbate is given by  due to the smaller mass of the dissociation products.

According to the equipartition theorem, the translational contribution to the energy is *E*_a,trans_ = *k*_B_*T*. For an ideal 2D gas, the entropy of the adsorbate is the sum of single-molecule (or single-atom) contributions, so we can use eq 18 without further considerations.
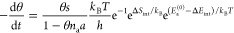
21Here, we have assumed non-dissociative adsorption (*N*_d_ = 1) since dissociative adsorption implies a strong interaction of the dissociation products with the surface, which we consider incompatible with a vanishing diffusion barrier. Equation 21 yields first-order desorption if the sticking coefficient *s* is given by
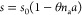
22where *s*_0_ is the initial (low-coverage) sticking coefficient.^40^

As expected, the activation energy (in the exponent of the last term of eq 21) comes out as −(*E*_a_^(0)^ − ). The pre-exponential factor is
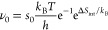
23If the initial sticking coefficient *s*_0_ at the desorption temperature is high (or unity), and neglecting the rotational and vibrational entropy contributions, the pre-exponential factor ν_0_ is in the order of 10^12^ to 10^13^ s^–1^. The term including Δ*S*_int_, the change of the vibrational and rotational entropies, is as expected from TST.^11^ Since the adsorbed molecules typically have less rotational freedom than those in the gas phase (for the ideal 2D gas, one might expect free rotation around a vertical axis only), there will be an increase of the prefactor by less than an order of magnitude for small molecules such as N_2_ or C_2_H_6_ at room temperature (these have *S*_rot_/*k*_B_ < 10 in the gas phase at 300 K).

In practice, the assumption of a flat potential energy surface, that is, negligible diffusion barriers, is rarely fulfilled. In most cases, the diffusion barriers between adsorption sites are much larger than *k*_B_*T* at the desorption temperature. This case will be discussed in the following two sections.

## 4 Case 2: An Ideal Lattice Gas

### 4.1 Basic Equations and the Prefactor

We now assume non-interacting adsorbate molecules at equivalent sites, separated by barriers higher than *k*_B_*T*, that is, a lattice gas. If the barrier is larger than 5–10 *k*_B_*T*, the harmonic oscillator model is a good approximation for the hindered translation; otherwise, a more elaborate calculation of the respective terms can be used.^34,41^ Except for the configurational entropy *S*_config_ of the adsorbate, we can treat the molecules as independent and simply use the entropy and enthalpy contributions per adsorbate molecule, as mentioned above. The configurational entropy per adsorption *site* is given by the equation for the mixing entropy. (We have a mixture of occupied and unoccupied sites; this expression is equivalent to the equation for *S*_config_ in ref (12).)
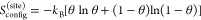
24For calculating μ_a_, the derivative d*S*/d*N* is required, see eq 4. The derivative, now with respect to θ, not *N*, since we use quantities per site or per adsorbed molecule, is given by^42^
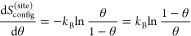
25It should be stressed that this is how *S*_config_ enters the equation for μ_a_; there is no term directly invoking *S*_config_ per adsorbed molecule because *S*_config_ is not proportional to the number of molecules. We note already at this occasion that the function in eq 25 multiplied by *T* (as it is when calculating μ) is the inverse function of the Fermi–Dirac distribution; the link to Fermi–Dirac statistics will become more clear in Section 5.1.

The chemical potential of the adsorbate is therefore given by
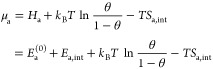
26From eqs 16 and 18, we obtain
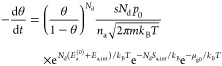
27
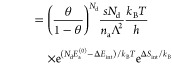
28with Δ*S*_int_ and Δ*E*_int_ defined by eq 19. We provide two versions of the equation, 27 for gases with μ_g0_ obtainable from tabulated values and 28 for other gases, where the energy and entropy difference of the internal degrees of freedom between the gas phase and the adsorbate must be calculated.

In the case of non-dissociative adsorption (*N*_d_ = 1), if the sticking coefficient is proportional to the number of free adsorption sites
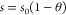
29that is, Langmuir adsorption, eqs 27 and 28 assume the shape of the Polanyi–Wigner equation for first-order desorption with an activation energy of −(*E*_a_^(0)^ − ). The pre-exponential factor is
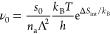
30As in the case of the adsorbate forming an ideal 2D gas (eq 23), the term containing Δ*S*_int_ is as expected from TST.^11^

Note that 1/*n*_a_ in eq 30 is the area per adsorption site; hence, *s*_0_/*n*_a_ can be seen as a “capture area” per free adsorption site: if each molecule impinging on a free adsorption site within this capture area gets bound as a stable adsorbate, and all the others get reflected, we obtain the sticking coefficient *s* as in eq 29. The first term in eq 30 is the ratio of this capture area and the square of the thermal de Broglie wavelength Λ. The latter provides a “natural” length scale relevant to the pre-exponential factor. The significance of Λ is also intuitively clear since Λ^2^ is essentially the area per microstate in a monatomic 2D ideal gas, cf. eq 20, and the number of microstates enters the entropy change in TST via Boltzmann’s entropy equation.

For dissociative adsorption of a diatomic molecule (*N*_d_ = 2), eq 27 or 28 results in second-order desorption if the sticking coefficient is given by
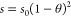
31This means, dissociative adsorption of a diatomic molecule leads to second-order kinetics if the sticking probability is proportional to the probability that *two given sites* (e.g., the two sites closest to the position of the incoming molecule) are unoccupied.

### 4.2 Direct Determination of the Adsorption Energy from TPD

In the simple cases of an ideal 2D gas or an ideal lattice gas with a single adsorption energy, the equation for the desorption rate, 16–18 can be rearranged to directly yield the adsorption energy *E*_a_^(0)^. For the case of an ideal lattice gas (eqs 27 and 28), this results in
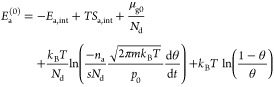
32
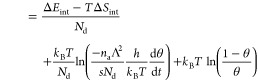
33For the determination of *E*_a_^(0)^ from eqs 32 or 33, the desorption rate dθ/d*t* must be known quantitatively. Under the assumption of an ideal lattice gas, these equations are valid at all coverages; we can also employ them at the temperature of the desorption peak (at the maximum of the desorption rate). In the following, θ_0_ is the initial coverage; it must correspond to the integral over the intensity *I* of the TPD spectrum.^3,6^ With β being the heating rate, this means
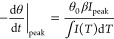
34where *I*_peak_ is the signal intensity measured at the peak and θ_0_ is the initial coverage. For first (second)-order desorption, simulated TPD spectra show that the coverage at the peak is about θ_peak_ ≈ 0.38 θ_0_ (0.52 θ_0_); this value should then be used in eqs 32 or 33. In these cases, simulated spectra also show that the integral in eq 34 can be expressed as the product of the peak height *I*_peak_ and the FWHM (full width at half-maximum, Δ*T*_fwhm_) of the peak, multiplied by 1.1. At the peak temperature, this yields for first-order desorption
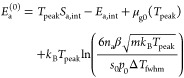
35
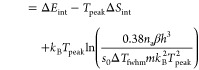
36For second-order kinetics (dissociation), the adsorption energy per atom can be determined via
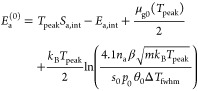
37When values in J/mol are desired, only the first *k*_B_ in eqs 35–37 should be replaced by the gas constant *R*, not the *k*_B_ in the argument of the logarithm.

These equations require knowledge of the initial sticking coefficient *s*_0_ at the adsorption temperature, the density of adsorption sites *n*_a_, and, for second-order desorption, the initial coverage θ_0_. These are only “weak” parameters (the prefactor scales with *s*_0_/*n*_a_, see eq 30). The relevance of the sticking coefficient will be discussed in Section 7.4. Concerning *n*_a_ and θ_0_, in many cases, the saturation coverage is known or can be estimated; otherwise, the gas can be dosed with a calibrated molecular beam^43^ or well-defined background pressure at low temperature, where the sticking coefficient is close to unity. For eqs 35 and 37, as mentioned above, the gas-phase chemical potential μ_g0_ at the reference pressure (10^5^ Pa) can be obtained from tabulated values.^28,29^ As will be discussed in Section 7.5, one can often neglect the internal degrees of freedom of the adsorbate, that is, the first two terms in eqs 35 and 37. For gases without tabulated μ_g0_ values, one can use eq 36 with the appropriate contributions for the internal degrees of freedom. Usually, the gas-phase rotation is the main contribution to Δ*E*_int_ and Δ*S*_int_ in eq 36 (see Section 7.5). The energies and entropies of the internal degrees of freedom, when not negligible, should be evaluated at *T*_peak_, the temperature of the desorption peak.

## 5 Distribution of Adsorption Energies

### 5.1 Basics and the Desorption Rate

On real-world surfaces (in contrast to perfect low-index single-crystal surfaces) or complex surface structures, it is common to have a lattice gas (well-defined adsorption sites separated by barriers larger than a few *k*_B_*T*) with a distribution of adsorption energies. In such a case, the configurational entropy of the adsorbate is no longer expressed by eq 24. This can be understood qualitatively: assuming a coverage θ that is not 0 or 1, as long as the low-energy sites are fully occupied, or as soon as the high-energy sites are all empty (because the molecules have desorbed from there), these sites do not contribute to the configurational entropy, and the entropy of the adsorbate will be lower than eq 24. The configurational entropy affects the TPD spectrum. Thus, the TPD spectrum of a surface with a distribution of adsorption energies is not simply the superposition of the TPD spectra corresponding to the different adsorption energies.

Since each site of a lattice gas can be occupied by zero or one molecule, assuming that the adsorbed phase is in equilibrium, we can treat it with Fermi–Dirac statistics (irrespective of the spin of the adsorbate atom or molecule).^16^ The adsorption sites then take the role of the microstates in Fermi–Dirac thermodynamics. We assume a continuous distribution of adsorbate energies ε, with a density ρ(ε), normalized such that
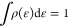
38Using Fermi–Dirac statistics, we could use eq 4 to determine the chemical potential of the adsorbed phase based on the enthalpy (energy) and the derivative of the chemical potential. There is an easier approach, however: since the thermodynamic quantities of a Fermi gas are usually expressed as a function of the chemical potential μ, we make use of the equation for a monatomic Fermi gas
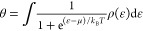
39This equation is usually written with a number of particles *N* at the left-hand side; in our case, we take particles per adsorption site θ in agreement with the normalization in eq 38. Since this equation is for a monatomic Fermi gas, μ does not contain the vibrational and rotational contributions to the entropy of the adsorbate, only the configurational part. Thus, μ_a_ = μ – *TS*_a,int_. In principle, the vibrational (and rotational, if any) energies *E*_a,int_ of the adsorbed phase should be included in the energy ε since there is no difference between the binding energy to the substrate (this can be considered a potential energy) and a vibrational energy of an adsorbate at this site (consisting of both, potential and kinetic energies). Then, ε in eq 39 would correspond to the energy of the adsorbate in the previous sections, ε = *E*_a_ = *E*_a_^(0)^ + *E*_a,int_, just that we now have a distribution of such energies. For simplicity, in the following, we will assume that the energy *E*_a,int_ of the interior degrees of freedom of the adsorbate (its vibrational modes) does not depend on the adsorption site and is just a constant offset to ε.

As discussed in the context of eq 15, the quantity of interest is usually *E*_a_^(0)^, not *E*_a_^(0)^ + *E*_a,int_, since *E*_a_^(0)^ describes the strength of binding. We therefore shift the variable of integration (the energy axis) in eq 39 by *E*_a,int_ to express θ via the distribution of the adsorption energies, ρ(*E*_a_^(0)^). If we add *E*_a,int_ to the expression for μ_a_ discussed above, we can leave the form of the equation unchanged
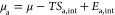
40
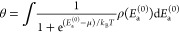
41

To determine the desorption rate for a given distribution of adsorption energies ρ(*E*_a_^(0)^) and a given coverage θ, we have to solve eq 41 numerically to obtain μ and then use eqs 3 and 40, which results in
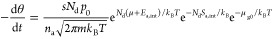
42
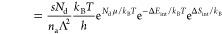
43Again, the two variants of the equation, 42 and 43, are for gases with tabulated values of μ_g0_ and other gases, respectively.

We can easily verify that eqs 42 and 43 are consistent with the equations in Section 4 by assuming a single value *E*_a_^(0)^ of the adsorption energy, which can be expressed using the Dirac δ distribution
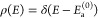
44Equation 41 then leads to
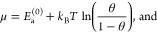
45
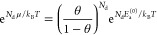
46Together with eqs 42 or 43, this directly yields eqs 27 or 28. Equation 45 is essentially the inverse of the Fermi–Dirac distribution (the fraction in the integral of eq 41); as mentioned earlier, it directly relates to the contribution of the configurational entropy of an ideal lattice gas in eq 26.

### 5.2 Calculation of the Energy Distribution from TPD Spectra

For the reverse direction, calculating the distribution of the adsorption energy distribution, we need a single spectrum at saturation coverage, which is defined as θ = 1 in accordance with eqs 38 and 39. We first have to determine μ for each point of the TPD spectrum (we solve eqs 42 or 43 for μ)
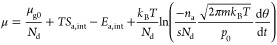
47
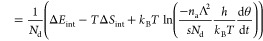
48With eqs 47 or 48, at each point of the TPD curve, *T*, μ, and θ are known (θ can be obtained from integration over the desorption rate; see also eq 34). Then, we have to find a distribution of adsorbate energies ρ(*E*_a_^(0)^) that fulfills eq 41 for the *T*, μ, and θ values at each point of the TPD curve.

First, let us assume that ρ(*E*_a_^(0)^) has only slow variations, that is, a very wide and smooth distribution of adsorption energies, resulting in TPD peaks that are many times wider than what would result from a single adsorption energy (eqs 27 and 28). Then, we can approximate the Fermi–Dirac distribution function (“Fermi function”) in eq 41 by a step function
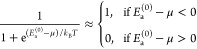
49This approximation allows us to directly determine the distribution of adsorption energies
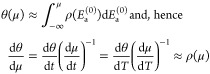
50Having calculated μ for each point of the TPD curve from eqs 47 or 48, this yields the ρ function, that is, the distribution of adsorption energies. The absolute value of dθ/d*t* can be obtained from the initial coverage θ_0_ in analogy to eq 34. Alternatively, since a constant scale factor of dθ/d*t* only results in multiplying ρ by the same factor, we can also normalize ρ according to eq 38.

The approximation in eq 49 neglects the thermal smearing in the conversion from ρ(ε) to the desorption rate, dθ/d*t*, caused by the integral in eq 41. To illustrate this issue, we differentiate eq 41:
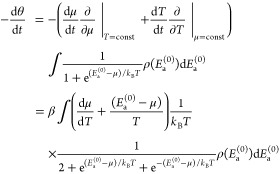
51

**Figure 1:**
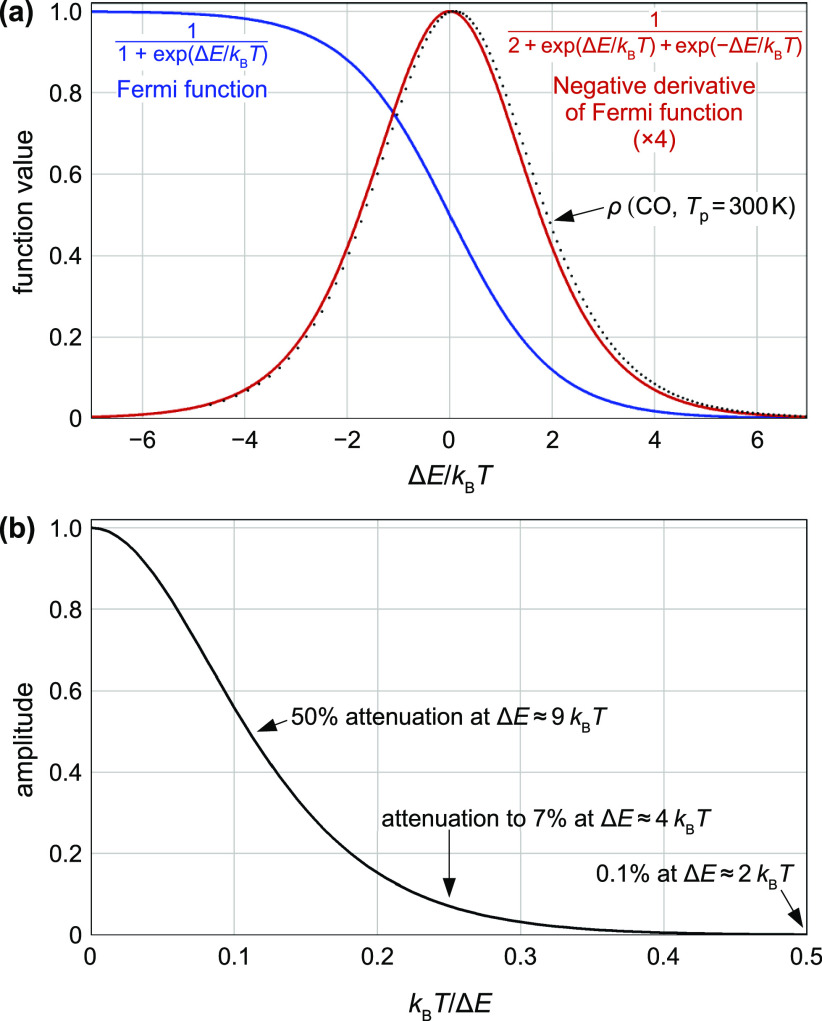
The Fermi function and its derivative. (a) Plots of the Fermi function and its negative derivative (the latter has been multiplied by 4 to better fit the *y*-axis range). The dots show the energy distribution calculated with eq 50 vs (μ − *E*_a_^(0)^)/*k*_B_*T*, from a CO TPD curve with a single *E*_a_^(0)^ = 105 kJ/mol (peak at *T* = 300 K; other parameters as in Section 7.1; also this curve is scaled vertically to fit the plot). This exemplifies that the output of eq 50 is close to a convolution of ρ(*E*_a_^(0)^) with the negative derivative of the Fermi function. (b) The Fourier transform of the negative derivative of the Fermi function can be used to estimate the energy resolution in a TPD experiment. Since the Fourier transform reaches a negligible value of 10^–3^ at *k*_B_*T*/Δ*E* = 0.5, it is impossible to resolve energies separated by Δ*E* = 2*k*_B_*T*.

This expression is very similar to a convolution integral. It would be a true convolution integral for constant dμ/d*T* and neglecting the variation of *T* appearing in various places in the integral. (The variation of *T* along the TPD curve is rather slow, compared with the changes caused by the variation of μ in the (*E*_a_^(0)^ − μ)/*k*_B_*T* term of the exponents.) The first term in the integral contains two summands: We find that the first one, dμ/d*T* dominates, it is typically in the order of magnitude of −50*k*_B_ at the peaks, whereas the second one is at most ±5*k*_B_ in the relevant range (where the derivative of the Fermi function has values sufficiently different from zero, see below). Thus, the desorption rate is approximately proportional to the convolution of the adsorption energy distribution ρ(*E*_a_^(0)^) with the negative derivative of the Fermi function (the fraction with 2 +... in the denominator of eq 51). This function is shown in Figure 1a; it is a bell-shaped curve, somewhat similar to a Gaussian distribution but with slower decay toward the sides [∼exp(−|*x*|) vs ∼exp(−*x*^2^)]. Convolution with it leads to smoothing of ρ(*E*_a_^(0)^). This means that the determination of the energy distribution ρ(*E*_a_^(0)^) from an experimental TPD spectrum is very similar to a deconvolution problem. Our computer program therefore uses a technique designed for deconvolution to calculate ρ(*E*_a_^(0)^), see Section 6.3.

### 5.3 Energy Resolution of TPD

The convolution-like step from the energy distribution to the desorption rate can be easily understood: at a given temperature, the desorbing molecules do not all come from sites with the same binding energy *E*_a_^(0)^. There will be some molecules with a weaker binding that have not desorbed yet, and a few molecules desorb although they bind more strongly than the average of the molecules desorbing at this temperature. In equilibrium, the occupation is given by the Fermi–Dirac distribution (Figure 1a), which will be shifted toward the left as the temperature increases during a TPD experiment. The difference of occupation between a temperature *T* and *T* + d*T* describes the molecules desorbing in the d*T* interval; it is given by the derivative of the Fermi–Dirac distribution. Strictly speaking, the shifted Fermi function at *T* + d*T* will also be slightly stretched along the energy axis since the temperature has increased, but this stretching will have an effect much smaller than the decrease of μ (the change of μ with temperature is very fast).

From a signal processing viewpoint, we can understand the convolution with the derivative of the Fermi function as a multiplication in Fourier space (convolution theorem). The high-frequency components of this convolution kernel are very low due to the rather slow and smooth variations of this function, see Figure 1b. This means that an exact solution of the deconvolution problem is not possible since the high-frequency information gets lost by multiplication with the low values of the Fourier transform of the kernel, and the deconvolution result would be excessively sensitive to noise in the experimental data. In other words, high-frequency components of ρ(ε), that is, variations over an energy scale smaller than a few *k*_B_*T*, cannot be resolved; about 93% of the modulation is already lost for variations with a periodicity of 4*k*_B_*T*, and 99.9% of the modulation gets lost at a periodicity of 2*k*_B_*T*. This makes it clear that variations of ρ(*E*_a_^(0)^) over such a small energy scale (5 kJ/mol or 0.05 eV at room temperature) cannot be resolved.

**Figure 2:**
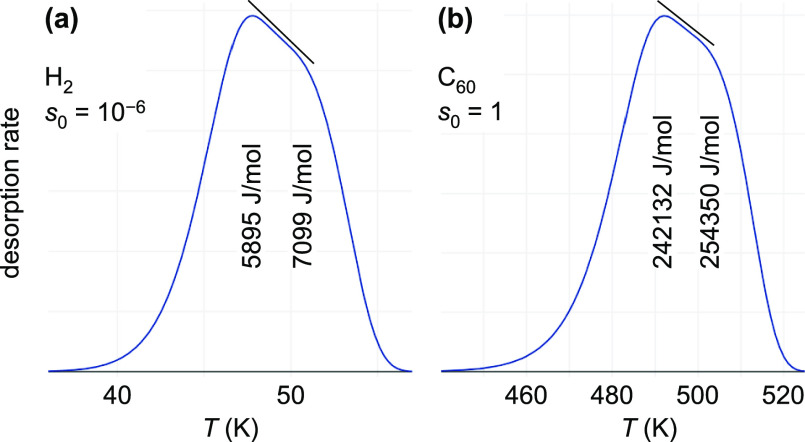
Calculated TPD spectra with two adsorption energies at the resolution limit. The straight lines above the peaks indicate that the spectra are at the limit between separate peaks (with a “dip” in between) and merging into a single peak. The calculations assume two sites, each with a density of 0.5 × 10^18^ m^–2^, but adsorption energies differ by 2.95 *k*_B_*T* (the |*E*_a_^(0)^| values are given inside the peaks). In both cases, Langmuirian sticking was assumed. For C_60_, *s*_0_ = 1 and the hypothetical case of no rotations of the adsorbed molecule and vibrational frequencies equal to the gas-phase molecule was assumed. For a single desorption peak at 500 K, this would lead to an extremely high pre-exponential factor of 5 × 10^25^ s^–1^ for C_60_, in contrast to only 6 × 10^8^ s^–1^ for the H_2_ case, where the low prefactor is due to the assumption of a low sticking coefficient (*s*_0_ = 10^–6^).

Assuming two adsorption energies, each containing the same coverage of molecules, that is, ρ(*E*_a_^(0)^) = δ(*E*_a_^(0)^ − *E*) + δ(*E*_a_^(0)^ − (*E* + *E*)), simulated TPD spectra (based on eqs 42 or 43) also show that the energy resolution is proportional to *k*_B_*T* for a very wide range of parameters. A single peak is seen when Δ*E* < 2.9*k*_B_*T*. In the limiting case, Δ*E* ≈ 2.95*k*_B_*T*, the TPD peak contains an almost linear section (with a nonzero slope, not horizontal, due to the nonlinear relation between μ and *T*, see Figure 2), and for Δ*E* > 3.0*k*_B_*T*, the linear section in the TPD curve turns into a region with a positive curvature, clearly indicating a double-peak structure. (We require a “dip” for resolution of two peaks, in analogy to the Sparrow resolution limit in optics. For closer peak separations, one may still infer the presence of more than one adsorption energy from the peak width or, if not too far below the resolution limit, from the peak shape, but we consider this below the resolution limit.) It is noteworthy that the ≈2.95*k*_B_*T* resolution limit applies not only for a wide range of adsorption energies (5–500 kJ/mol) but also for extremely different entropy contributions and different coverage dependence of sticking (Langmuirian or constant). In our test cases, due to the assumption of widely different sticking coefficients, densities of adsorption sites, and entropy contributions, a single desorption energy would lead to a peak FWHM between 3.7 and 27% of the peak temperature; nevertheless, the energy resolution remains essentially the same in terms of *k*_B_*T*.

## 6 Computer Program for TPD Calculations

### 6.1 Overview

**Figure 3:**
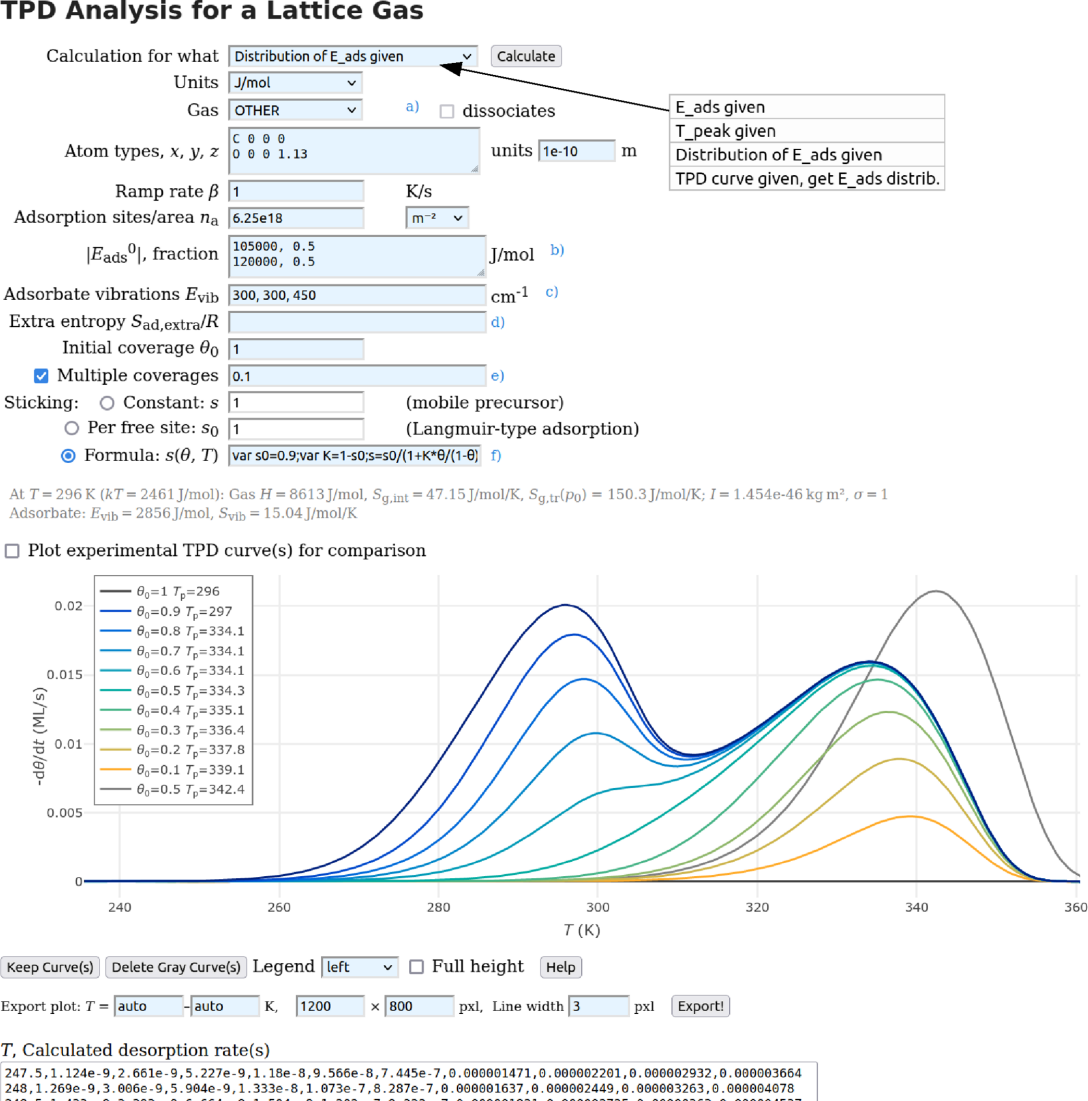
Screenshot of the computer program. Here, it is assumed that half of the adsorption sites have an adsorption energy of 105 kJ/mol, the other half 120 kJ/mol. The molecule is CO, not using the tabulated values for calculating μ_g0_ but rather the atomic coordinates given in Å units, as also possible for arbitrary molecules (gas type “other”). Using the tabulated data with gas type “CO” yields the same TPD spectra within the line width of the plot. The sticking probability *s* is described by a custom formula.^46^ The gray curve in the plot is the result of a previous calculation (assuming an adsorption energy of 120 kJ/mol at all sites); this curve had been saved with “Keep Curve(s)”.

The Supporting Information contains a computer program for TPD simulation of the lattice gas model based on eqs 27 and 28 for a single adsorption energy and eqs 42 and 43 for the case of a distribution of adsorption energies.^44^ The program was written in JavaScript and runs in a web browser.^45^ This provides an easy way of programming the user interface as an HTML input form (Figure 3) and ensures compatibility with all computing platforms. The program works with Langmuir-type sticking (i.e., first- or second-order desorption), coverage-independent (constant) sticking, as well as other functional forms of *s*(*T*, θ) provided by the user (e.g., Kisliuk’s equation^46^ or expressions taking detailed geometric consideration into account^22^).

The program includes the tabulated *H* and *S* values^28,29^ needed to calculate μ_g0_ for a few common gases (H_2_, D_2_, N_2_, O_2_, H_2_O, D_2_O, CO, CO_2_, and CH_3_OH).^47^ For other molecules, the user can enter the atom types and coordinates to calculate the rotational entropy in the gas phase; this is shown in Figure 3 for CO (this molecule would also be available as a pre-defined gas with tabulated values; the results for the desorption peak temperature are identical within 0.1 K). For user-defined molecules, the input format for the atom type and *x*, *y*, and *z* is the same as in the body an *XYZ* file used for molecular rendering. (*XYZ* files are available for a large number of molecules, e.g., in the library of the IQmol molecular editor and visualization package.^49,50^) For these molecules, the program calculates the gas-phase moments of inertia and the σ value required in eqs 11 and 13 from the coordinates and atomic masses.^51^ If vibrational modes are known, the user can enter them manually (vibrations with *h*ν > 2*k*_B_*T* can be considered frozen modes and neglected). Currently, our code provides no easy way of taking into account that low-frequency hindered translation modes (which can have a non-negligible influence on the result) can become stiffer with increasing coverage due to repulsion between the adsorbates, except for formulae entered in the “extra entropy” field.

When comparing simulated and experimental TPD curves, it should be taken into account that the ionization probability in a mass spectrometer depends on the velocity of the molecules, which determines the time they spend in the ionization region. If the desorbing molecules directly enter the mass spectrometer, without being reflected by any surface, molecules desorbing at higher temperatures will usually have a higher velocity and therefore lower ionization probability. Our program therefore contains an option to scale the intensities of experimental TPD spectra (plotted for comparison with the calculated ones or taken as an input for the determination of the adsorption energy distribution, see Section 6.3) with a  factor. This correction is required mainly for TPD spectra encompassing a wide temperature range (multiple desorption peaks).

### 6.2 Integration of the Desorption Rate

Simulating a TPD curve requires integrating the desorption rate over the temperature to obtain the coverage θ(*T*). We found that numeric integration works best when not calculating dθ/d*T* but rather d ln θ/d*T* and then iterating over temperature steps Δ*T*:
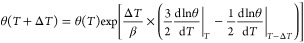
52The difference in the inner bracket is an extrapolation of d ln θ/d*T* to *T* + Δ*T*/2, taking changes of d ln θ/d*T* with a linear temperature dependence into account (one can consider it the predictor step of a predictor–corrector method). Especially in the rising and falling tails of a TPD curve, d ln θ/d*T* is almost linear, in contrast to the highly nonlinear dθ/d*T*; thus, the linear extrapolation works better with d ln θ/d*T*. With this method, it would be possible to use rather coarse temperature steps (about 1% peak height accuracy and negligible error of the peak temperature with 10 steps per FWHM); nevertheless, we use finer steps to be on the safe side. We apply this method of integration only to calculated desorption rates, not to the experimental ones (the latter are used as input to calculate the *E*_a_^(0)^ distribution in Section 6.3) because experimental data contain noise. Applying a nonlinear function to noisy data would introduce a bias when integrating. (This is the same problem as when averaging over a nonlinear function of noisy data.)

### 6.3 Calculation of the Adsorption Energy Distribution

As described in Section 5.2, with an appropriate conversion between adsorption energies and temperatures *T*, given by eqs 41, 47, and 48, and θ(*T*), the TPD curve can be approximated by a convolution applied to the distribution of adsorption energies ρ(*E*_a_^(0)^), see eq 51. We can therefore employ the Richardson–Lucy (R–L) method^52,53^ to determine ρ(*E*_a_^(0)^) from a TPD spectrum at saturation coverage. The R–L method is well suited as it is designed for data with Poisson noise, which is typical for mass spectrometer data. Although originally devised strictly as a deconvolution method, we find that the R–L method can also be applied to our case, where the forward transformation (from ρ(*E*_a_^(0)^) to dθ/d*T*) is only approximately described by a convolution. The R–L method works by iterations, where correction factors for each data point are applied to the result of the previous step:
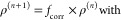
53
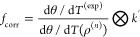
54Here, −dθ/d*T*^(exp)^ is the experimental desorption rate and −dθ/d*T*(ρ^(*n*)^) is the rate calculated for the distribution of adsorption energies in iteration step *n* (the forward transformation; in the standard applications of the R–L method, this would be the result of a convolution of ρ^(*n*)^ with a given kernel). The ⊗ symbol denotes convolution with a kernel *k*′. In the standard R–L method, *k*′ would be the mirror-reversed kernel of the forward convolution applied to ρ^(*n*)^. In our case, the forward transformation is close to a convolution with an almost-symmetrical, bell-shaped function decreasing monotonously at both sides (Figure 1a; there is a slight additional skew because the *x*-axis is *E*_a_^(0)^, not Δ*E*/*k*_B_*T*). In such a case, the choice of *k*′ is not critical; the convolution with *k*′ could even be omitted, though this would lead to increased amplification of experimental noise. In our program, we use a Hann window function (i.e., the cos^2^ function in the ±π/2 range) for *k*′, with a FWHM of 2*k*_B_*T*. This width provides a good compromise between computational speed (faster convergence of the R–L method for smaller FWHM) and suppression of high-frequency noise (better with higher FWHM). We start the iterations with ρ^(0)^ taken from eq 50. For noisy data, this curve is rather noisy and therefore slightly smoothed to reduce the influence of the noise. Since the R–L iteration steps do not exactly preserve the normalization of ρ(*E*_a_^(0)^), we have to normalize ρ according to eq 38 between the steps.

**Figure 4:**
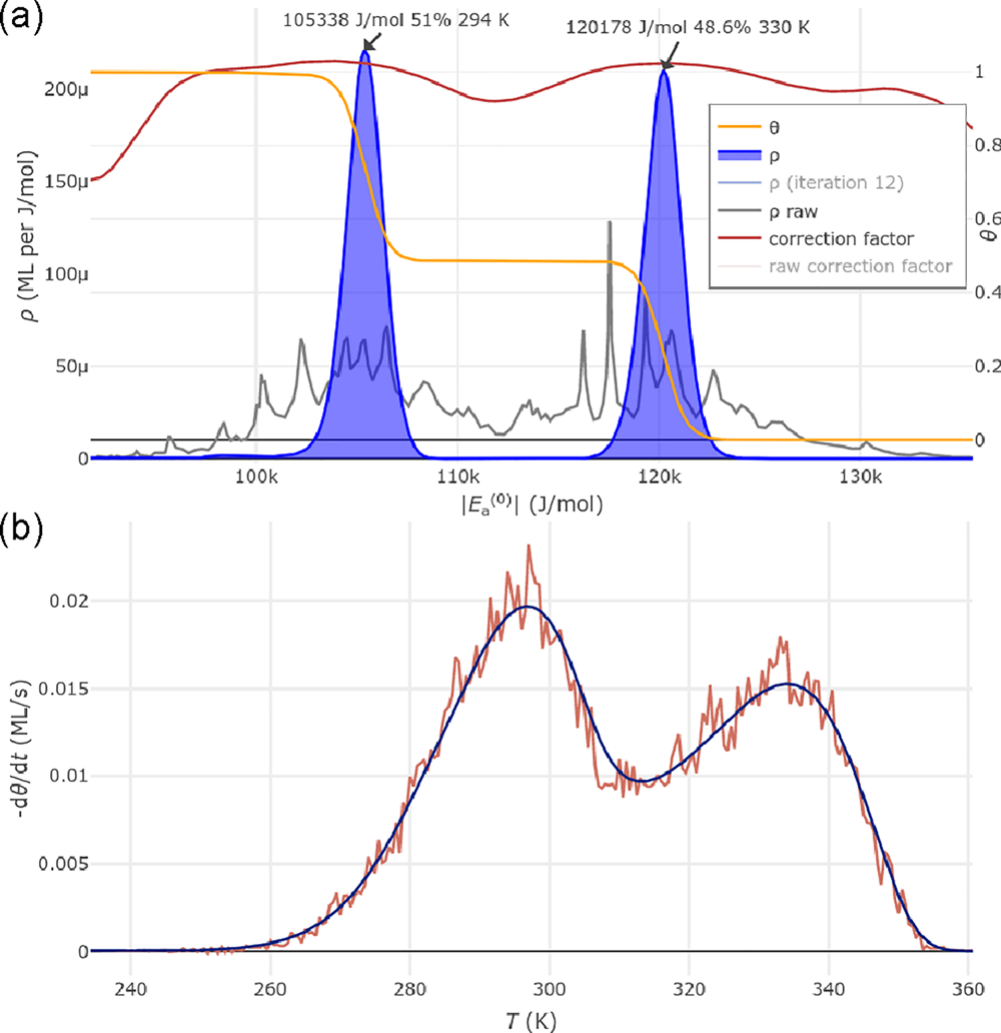
Screenshot of the computer program showing a calculation of the adsorption energy distribution. The calculated TPD curve for θ = 1 in Figure 3, assuming two adsorption sites with |*E*_a_^(0)^| = 105 and 120 kJ/mol, is used as the input, but with added noise [red curve in panel (b)]. Apart from the finite energy resolution, the calculated energy distribution in (a) comes close to the original *E*_a_^(0)^ distribution. Panel (b) also shows the TPD curve obtained with the calculated energy distribution (dark blue). The temperatures in the peak labels in (a) are not TPD peak temperatures, but the temperatures corresponding to the μ = *E*_a_^(0)^ values in the plot according to eqs 47 and 48; these are for assignment of the adsorption energies to features in the TPD spectra, but not strictly identical to the peak temperatures. Some of the curves in the ρ(*E*_a_^(0)^) plot (ρ_raw_, correction factor) are related to the R–L algorithm and displayed in “expert mode” only, and two curves have been hidden (light gray in the legend).

As discussed in Section 5.3, an exact solution of the deconvolution problem is not possible. Due to the strong decay of the Fourier transform of the (approximate) kernel, see Figure 1b, rapid variations of ρ(*E*_a_^(0)^) with the energy cannot be resolved; attempts to do so would amplify even the slightest experimental noise to unacceptable values. In a standard deconvolution method, this could lead to negative values of ρ(*E*_a_^(0)^). The R–L method never creates negative values; for a high number of iterations, the experimental noise will instead lead to the appearance of isolated peaks in ρ(*E*_a_^(0)^) even when ρ(*E*_a_^(0)^) is a broad distribution. To mitigate this overfitting, our program contains an algorithm that applies weaker corrections (correction factors closer to unity) in regions where the correction factors show no or only a gentle dependence on *E*_a_^(0)^. Refinement of narrow peaks in ρ(*E*_a_^(0)^) will be less affected because there the correction factors vary more rapidly. Figure 4a shows an example of a calculated adsorption energy distribution (blue) using the default settings for the R–L method in our program. The noisy, gray curve shows the raw (unsmoothed) ρ values from eq 50. In the example of Figure 4, a higher number of iterations would lead to narrower peaks in ρ(*E*_a_^(0)^) (the input data are based on two sharp adsorption energies), but the fit of the input spectrum would be only marginally improved. If desired, the user can select the “expert mode” checkbox and enter the number of iterations and a “noise suppression” parameter that weakens the correction in regions of slow variations (as described above).

Each R–L iteration requires calculating a TPD spectrum as the forward transformation, which involves some computational effort (including the numeric solution of eq 41 for each point of the experimental TPD spectrum). It is common to accelerate the R–L method.^54^ Our algorithm uses an exponent for the correction factor as suggested in ref (55); in contrast to a linear amplification of the correction, this guarantees that the ρ values remain positive. We avoid stability problems by adjusting the exponent: the smaller the change of the correction factors from one iteration to the next, the higher the value of the exponent. The gain in computing speed is about a factor of 3–4.

As a side note, the conversion between temperatures *T* and the corresponding *E*_a_^(0)^ values (the μ in eqs 47 and 48), which forms the basis of calculating the energy distribution, is not as straightforward as it may seem. Experimental desorption rates contain noise, and the appearance of the desorption rate in eqs 47 and 48 leads to noise in the *E*_a_^(0)^ values, which usually makes them non-monotonous. Of course, the molecules with weakest binding desorb first; thus, the true μ or *E*_a_^(0)^ values of the desorbing molecules must monotonously decrease with increasing temperature. (We use the convention that more negative *E*_a_^(0)^ means more strongly bound molecules.) Our code contains several provisions for “cleaning” the *E*_a_^(0)^ values corresponding to the temperatures of the desorption curve, starting with the elimination of negative experimental rates (which can occur if background subtraction was applied to the experimental data) and slight smoothing of the desorption rate. Then, median and three-point moving-average filtering is applied to the raw *E*_a_^(0)^ values. Finally, regions of non-monotonicity of *E*_a_^(0)^ are replaced by linear interpolation between the nearest points that yield a monotonous curve, followed by another moving-average run. These steps of data cleaning only affect the selection of the points on the *E*_a_^(0)^ axis. The ρ(*E*_a_^(0)^) values are unaffected as long as the *E*_a_^(0)^ points are dense enough (i.e., if the temperature steps are sufficiently small, which is usually the case).

The output lists of the program allow using the results as an input: one can copy and paste the list of temperatures and desorption rates of a simulation with a fixed adsorption energy or a given distribution of adsorption energies and provide it as an “experimental” input to determine the distribution of adsorption energies from it. (When calculating the TPD curve, “multiple coverages” should be deselected, to avoid using a low-coverage curve as an input, and for the (pseudo-)experimental curve, one should deselect “ionization probability correction”.) This can be useful to explore the possibilities of the program. One can also verify that the coverage-dependent TPD curves for adsorption with a precursor and a single adsorption energy can be almost perfectly reproduced by assuming Langmuirian sticking and a distribution of adsorption energies (see Section 7.2).

If the input is a TPD curve for a single adsorption energy or a distribution containing very sharp (δ-like) peaks, one should not expect similarly sharp peaks in the energy distribution calculated with the same parameters as the input curve. This is due to the finite energy resolution of TPD (Section 5.3); the R–L algorithm starts from a broadened distribution, and refinement is limited as otherwise the noise sensitivity would be too high. (To some degree, this can be controlled in the “expert mode.”) Figure 4 shows an example of such a calculation.

Alternatively, one can also copy the energy distribution obtained in such a calculation and paste it as an input to the “|*E*_ads_^(0)^|, fraction” field of a TPD simulation with a given energy distribution. This may be helpful to examine the impact of various parameters on the TPD spectra. (The extra data columns present in the output and pasted into the “|*E*_ads_^(0)^|, fraction” field are ignored.)

## 7 Discussion

### 7.1 Pre-exponential Factor

Let us first consider the most simple case, first-order desorption. The potential energy landscape for most adsorbates is corrugated; if these energy variations are well above *k*_B_*T*, we have to assume a 2D lattice gas. Even for van der Waals-bound small molecules on close-packed metal surfaces, where very low diffusion barriers are expected, experiments indicate ratios between the diffusion barrier and the desorption barrier of ≈0.1–0.3.^56−58^ Since the desorption barrier is typically about 30–40*k*_B_*T*_peak_,^60^ most diffusion barriers are at least ≈3*k*_B_*T* at the desorption temperature, which is in the regime of a lattice gas.

Equation 30 shows that the pre-exponential factor for a lattice gas with first-order adsorption is usually not in the order of *k*_B_*T*/*h* or 10^13^ s^–1^, as one might naively expect given the usual prefactors for many thermally activated processes such as diffusion. As a simple example, let us consider CO with adsorption sites in a square lattice with 4 Å side length, which is a typical CO–CO distance, and Langmuir adsorption (leading to first-order desorption), with an initial sticking coefficient of unity. Under these assumptions, and ignoring Δ*S*_int_ contributions, a TPD peak appearing at 300 K (with β = 1 K/s) would have a prefactor of 2.8 × 10^15^ s^–1^. Including the rotational degrees of freedom of the gas phase but neglecting vibrations of the adsorbate increases the prefactor to 8 × 10^17^ s^–1^. The vibrational modes of the adsorbed CO will reduce this: assuming an isolated binding site, two wagging modes of 300 cm^–1^ and a vertical translation of the molecule at 450 cm^–1^ reduce the prefactor to 1.3 × 10^17^ s^–1^. On surfaces such as single-atom catalysts, where the density of binding sites is low,^61^ assuming a “capture area” larger than a typical atomic size (some inverse spillover to the binding site), the prefactor would be even higher. For CO bound on metal surfaces (in contrast to non-metals with isolated metal sites binding the CO), the lowest-frequency modes are usually hindered translation modes; according to DFT calculations, these can be as low as ≈50 cm^–1^ (ref (62); lower frequencies would be usually associated with diffusion barriers that are too low for application of the lattice gas model with a harmonic oscillator potential). Adding two 50 cm^–1^ modes affects the prefactor strongly through their high vibrational entropy and reduces it by about 2 orders of magnitude to 10^15^ s^–1^. It should be noted, however, that DFT most likely underestimates the relative stability of the stable adsorption site (on-top) in this case, similar to CO/Pt(111);^22,63^ this would also lead to an underestimation of the frequency of the hindered translation and the prefactor. On the high-frequency side, modes with *h*ν > 2*k*_B_*T* have hardly any effect; for example, the C–O stretch mode (≈2000 cm^–1^) is frozen at room temperature and plays no role.

One reason for the prefactor being much higher than the naive estimate of ≈10^13^ s^–1^ is the fact that the thermal de-Broglie wavelength Λ is typically small compared to molecular dimensions or the distance between neighboring sites in a 2D lattice gas. For CO at 300 K, Λ ≈ 20 pm. It comes close to molecular sizes only for very light species or very low temperatures: For an H atom, Λ ≈ 100 pm at 300 K. The other reason for the high prefactor (except for monatomic gases) is the rotations in the gas phase that are not present in the adsorbate. Again, the increase of the prefactor is substantially less for H_2_ (due to the low moment of inertia); there, the internal degrees of freedom of the gas phase increase the prefactor by only a factor of ≈5 at room temperature.

Therefore, except for hydrogen, a prefactor as low as 10^13^ s^–1^ should be considered the exception rather than the rule for all cases where the adsorbate can be described as a lattice gas (if diffusion barriers are at least a few *k*_B_*T*). Prefactors of 10^13^ s^–1^ or below should occur only if the sticking coefficient is very low. For molecules larger than CO, the thermal de-Broglie wavelength will be shorter and the rotational inertia will be higher; thus, one should expect prefactors above 10^17^ s^–1^. One of the largest molecules that can desorb without fragmenting is probably C_60_;^64^ due to its large mass and high rotational inertia, the prefactor would be ≈10^26^ s^–1^ for desorption at 450 K when vibrations of the adsorbed molecules play no role and the molecules are trapped in well-defined sites with 2 nm spacing. This will be reduced by a several orders of magnitude when taking the vibrational entropy of the adsorbate into account. Thus, we consider ≈10^23^ s^–1^ the upper limit of prefactors that can be encountered for first-order desorption.

We should finally mention that the pre-exponential factor has some temperature dependence. Most TPD analysis methods consider a temperature-independent value. The temperature dependence of the prefactor (mainly through the entropy of rotation in the gas phase and vibrations of the adsorbate) will then influence the calculated adsorption energy. Fortunately, the errors caused by the temperature dependence of the entropy are not very large. For the example of CO at 300 K, when assuming Langmuirian sticking, a leading edge analysis of the TPD peak calculated by our program (which includes the temperature dependence of the entropy terms) leads to a 6% error in the energy when no adsorbate vibrations are considered. In the case of a severe influence of adsorbate vibrations discussed above (one mode with 450 cm^–1^ and two mode pairs with 300 and 50 cm^–1^ each), the error caused by neglecting the temperature dependence of the prefactor is close to the vibrational energy of the adsorbate (7.35 kJ/mol); thus, the leading edge analysis happens to yield the *E*_a_^(0)^ value within a percent, in spite of overestimating the prefactor by almost an order of magnitude.

### 7.2 Desorption Orders and Precursor-Mediated Adsorption

Our analysis clearly outlines the conditions for first- and second-order desorption: for non-dissociating adsorbates with well-defined adsorption sites (a lattice gas), first-order kinetics occurs if the sticking coefficient follows eq 29, that is, the Langmuir model for sticking. In the case of dissociation into two equivalent fragments, second-order kinetics occurs for a lattice gas with sticking coefficients in the form of eq 31. For these cases, as well as for the case of an ideal 2D gas with an appropriate coverage dependence of *s*, one can also define coverage-independent pre-exponential factors, and they can be described by the Polanyi–Wigner equation, eq 1. In the following, we will discuss two examples where this is not the case.

As mentioned above, for molecules dissociating into two equivalent fragments, our approach directly yields second-order kinetics for the case of Langmuir-like sticking requiring two specific sites to be free, as expressed by eq 31. It is known that dissociative adsorption of single-element diatomic molecules does not always show second-order TPD spectra. A well-known example is H_2_/Si(100), exhibiting first-order TPD spectra,^65^ possibly except for low coverages.^66^ In this case, the desorption process is very complex; the sticking coefficient is very low and has a non-trivial dependence on temperature and coverage. Since the sticking coefficient *s* appears as a factor in eq 28, its coverage dependence can influence the apparent desorption order.

**Figure 5:**
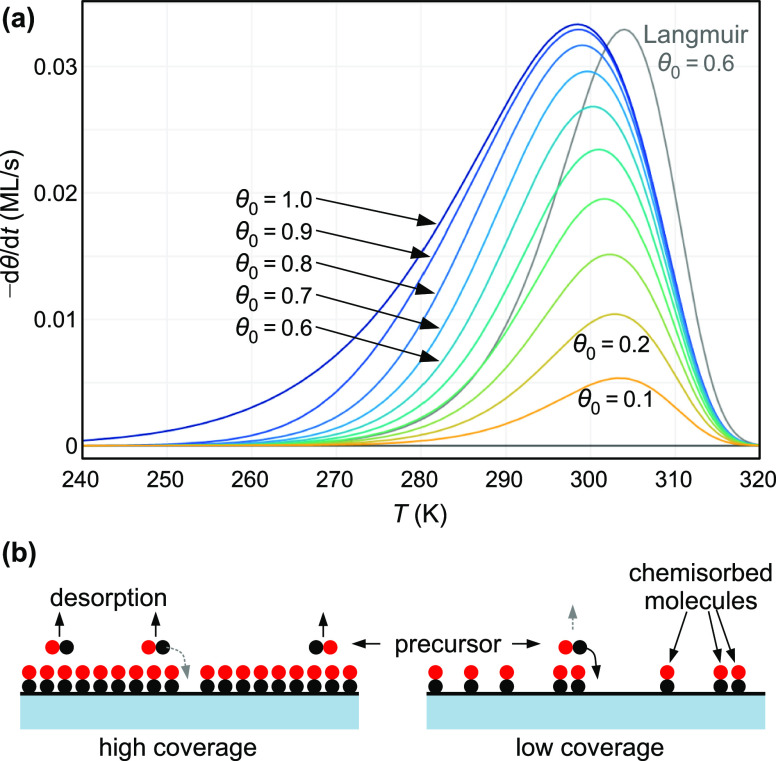
Adsorption with an extrinsic precursor. (a) Calculated TPD spectra for CO, for initial coverages up to θ_0_ = 1 in increments of 0.1, assuming a precursor model with a constant sticking coefficient *s* = 1. For comparison, a curve for Langmuirian sticking with θ_0_ = 0.6 and *s*_0_ = 1 is shown in gray. Parameters assumed: *E*_a_^(0)^ = 105 kJ/mol, β = 1 K/s, *n*_a_ = 6.25 × 10^18^ m^–2^, two modes with 300 cm^–1^ each (wagging), and one vibration mode with 450 cm^–1^ (vertical). (b) Schematic view of desorption in the presence of a precursor state. At coverages approaching unity, a molecule in the weakly bound precursor state will desorb with high probability, whereas at low coverages, it will usually fall back to the chemisorbed state and the precursor does not enhance the desorption rate. The density of precursor molecules is strongly exaggerated in the drawing; in reality, it is extremely low.

If the sticking coefficient is constant, for example, close to unity in the case of a mobile (extrinsic) precursor for adsorption, eq 28 yields a coverage-dependent pre-exponential factor. This leads to a deviation from first-order desorption, with the desorption peak shifting to lower temperatures with increasing coverage (Figure 5). The largest difference between the *s* = 1 case and first-order desorption occurs at coverages very close to 1, where the desorption rate is much higher in the presence of a precursor. Considering thermal equilibrium, as outlined in Section 2, the adsorption and desorption rates are directly linked (reversibility), see eq 2. For adsorption, a large difference between Langmuirian sticking and the precursor model is expected, as the incoming adsorbate can arrive anywhere on the surface and find one of the few empty sites by diffusing as a precursor. For desorption, detailed balance requires that the reverse process is also likely: at high coverage, the molecules, once they have reached the precursor state, can leave the surface everywhere, while the probability to find an empty site and fall back into the chemisorbed state is rather low, see Figure 5b. The assumption of constant sticking also leads to desorption peaks that are substantially wider than true first-order peaks:^67^ at θ_0_ = 1, the FWHM of the peaks in Figure 5a is about 65% larger than that for a first-order peak (Langmuirian sticking) with the same adsorption energy. For θ_0_ = 0.5, the peak is still 20% wider than a first-order peak would be.

Based on a conventional TPD analysis, a set of curves as in Figure 5a would be interpreted as a coverage-dependent adsorption energy, either caused by adsorbate–adsorbate repulsion when approaching saturation coverage or by a distribution of adsorption energies. Based on TPD data alone, there is no possibility to distinguish between the case of precursor-mediated sticking and the two other possibilities. (This highlights the importance of measuring the sticking coefficient vs coverage at a temperature close to the desorption temperature for a correct interpretation of the data; see also Section 7.4.)

It should be emphasized that Figure 5a was calculated for a single adsorption energy, independent of coverage and adsorption site. The coverage-dependent peak positions and the long tail toward the left for θ_0_ → 1 are the consequence of non-Langmuirian sticking. Whenever these features are found, one should consider the possibility of a precursor with non-Langmuirian sticking, where first-order TPD theory does not hold. On the other hand, if the experimental peak is as sharp as predicted by our model for Langmuirian sticking, and attractive interactions between adsorbates are unlikely (these can also lead to sharper peaks than in our model), this provides clear evidence against a strong influence of a precursor on sticking at the desorption temperature.

It has been shown long ago that conventional TPD analysis based on peak shape and/or width or leading edge analysis applied to a case of precursor-mediated sticking can result in large errors.^68^ Using the calculated TPD curve for θ_0_ = 1 in Figure 5a and assuming a first-order peak, leading edge analysis as well as the method of ref (8) gets only slightly more than half the correct value for the adsorption energy (≈54 instead of 105 kJ/mol). For θ_0_ = 0.5, these methods yield the correct adsorption energy within a few percent; in this case, the sticking does not differ so much from Langmuir adsorption. Using the peak width^7^ of the θ = 1 curve to calculate the pre-exponential factor is not much better than leading edge analysis (63 kJ/mol). This problem of incorrect desorption energies obtained by a classical TPD analysis is not purely hypothetical. Such a case was reported for dissociative adsorption of H_2_/Rh(100), where the analysis of the peak shape yields a desorption energy of 0.87 eV/molecule at low coverage, further decreasing with coverage, accompanied by a change of the apparent pre-exponential factor by several orders of magnitude.^69^ The accompanying changes of the apparent prefactor and adsorption energy values without a large change of the temperature of the TPD peak are known as the “compensation effect”;^70,71^ it was already noted in ref (69) that this can be an artifact of a TPD analysis assuming the validity of the Polanyi–Wigner equation where it is not justified. Analysis of these data based on the works of Payne and Kreuzer^17^ (essentially the approach also used by us) and with the experimentally determined (almost coverage-independent) sticking probability was compatible with the |*E*_a_^(0)^| value from density functional theory calculations (1.14 eV/molecule).

### 7.3 Activated Adsorption

For the case of activated adsorption, that is, a barrier Δ*E*_ad_ for the incoming molecule upon adsorption, the desorbing molecule has to overcome that barrier in addition to the adsorption energy. Our assumptions do not exclude such a case; nevertheless, the exponent in eqs 21, 27, and 28 contains *N*_d_*E*_a_^(0)^ – Δ*E*_int_, the difference of the energies between the gas phase and the adsorbed phase, irrespective of the adsorption barrier Δ*E*_ad_. At first glance, one would expect an Arrhenius term with the full height of the desorption barrier, including the additional Δ*E*_ad_, in the exponent of these equations. In such a case, the sticking probability is less than unity, however, and depends on the temperature of the incoming gas. Since our calculations are based on equilibrium thermodynamics, where the temperature of the gas and the sample must be the same, we have to use the sticking probability *s* as a function of (gas and surface) temperature. Thus, the adsorption barrier enters the equations via *s*(*T*), and it will lead to a stronger temperature dependence of the desorption rate than the term with *N*_d_*E*_a_^(0)^ – Δ*E*_int_ in the exponent; an additional Arrhenius term from the sticking probability *s* will appear. In such a case, our equations for the pre-exponential factor, eqs 23 and 30, contain terms that will enter the exponent, not the prefactor, and the prefactor in the conventional sense is not described by these equations.

In the case of activated adsorption, it would be required to determine the sticking coefficient *s* for molecules with the same energy distribution as in a gas at the desorption temperature. In practice, this may be difficult; thus, our method will be usually restricted to non-activated adsorption. Since other methods of TPD analysis do not yield the adsorption energies (but rather the desorption barrier) for activated adsorption, techniques beyond standard TPD, for example, true equilibrium measurements (adsorption isotherms or isobars) or more advanced methods,^72^ will be more suitable for this case.

### 7.4 Sticking Probability

The sticking probability *s* occurs in all our analysis; in the desorption rate, it enters the pre-exponential term. Therefore, an error in the value of *s* has essentially the same implications as an error of the pre-exponential factor in a conventional TPD analysis based on the Polanyi–Wigner equation, that is, it only weakly influences the analysis. Thus, if the value of *s* is guessed incorrectly by a factor of 2, this affects the peak temperature or the adsorption energy by only ≈2%. In TPD setups using a molecular beam for dosing, it is possible to measure the coverage-dependent sticking probability by the King–Wells technique.^73^ In the ideal case, the sticking measurement should be done at the temperature of the desorption peak since our analysis is based on the equilibrium thermodynamics at the desorption temperature. In practice, it may be easier to measure *s* at a temperature where the desorption rate is low, that is, slightly below the peak; this should be sufficient if *s* depends only weakly on *T*, which is usually the case. In the case of a mobile precursor, a measurement of the sticking probability at low temperatures will usually find a coverage-independent sticking probability close to unity, while the precursor might be bound too weakly to play a role at the desorption temperature. This might be especially true for a (model) catalyst with sparse metal adatoms or small clusters on an oxide support, where CO will weakly adsorb on the oxide and spill over to the metal at low temperatures only. A sticking measurement slightly below the TPD peak will be acceptable since the lifetime of the precursor molecules on the substrate will have a much weaker temperature dependence (lower desorption barrier) than desorption from the chemisorbed state.

### 7.5 Internal Degrees of Freedom (Rotations and Vibrations)

Using our equations requires the entropy of the adsorbed molecule to be known, including the entropy contributions of molecular vibrations. For determining the binding energy *E*_a_^(0)^, the vibrational energy also plays a role (this term is usually neglected in TPD analysis; for accurate comparison with DFT-calculated energies, it should be included). Fortunately, for small molecules, these terms have usually a rather small influence on the result. For our CO test example (Figures 3–5), a single mode with 300 cm^–1^ (37 meV) affects the calculated adsorption energy by 0.6% for a peak at 300 K and 1.4% for a 600 K peak. In the case of dissociative desorption of H_2_ on Rh(100) mentioned in Section 7.2,^69^ the calculated vibration modes of ≈90 (vertical) and ≈50 meV (parallel to the surface, two modes)^69^ influence the calculated adsorption energy by slightly less than 2%. As mentioned in Section 7.1, the impact of low-frequency modes is more substantial. In our above example of a CO TPD peak at 300 K, neglecting two hindered-translation modes with 50 cm^–1^ each would lead to an underestimation of |*E*_a_^(0)^| by 7%.^74^

In some cases, the value of the diffusion barrier for the molecule may be known, for example, if calculated energies in the stable site (potential-energy minimum) and the site of the transition state for diffusion are available.^75^ In such a case, we can get a guess for the vibrational frequency of the hindered translation by assuming a sinusoidal potential energy between two adjacent energy minima,^41^
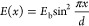
55where *d* is the distance between two adjacent minimum-energy sites and *E*_b_ is the energy barrier for diffusion, that is, the energy of the transition site minus the energy at the minimum. Assuming that the barrier is substantially higher than *h*ν, the vibrational frequency of the hindered translation can be calculated as^34^
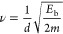
56Since the hindered translation modes are often those with the lowest frequency, they have the highest impact on the vibrational entropy, so eq 56 is useful to estimate the impact of the adsorbate vibrations on the result.

Large molecules have a large number of vibrational degrees of freedom; in many cases, the frequencies of the eigenmodes are not known. For a selection of molecules, mainly alkane chains, it has been argued that the entropy of the internal degrees of freedom of the adsorbed molecules is about ≈70% of the gas-phase values.^76^ This analysis was based on an evaluation of TPD prefactors assuming a 2D ideal gas for the adsorbate, which might be not too far from reality for van der Waals-bound alkanes (though not for all cases considered in ref (76)), but even for alkanes, the diffusion barriers might be too high for assuming an ideal 2D gas.^57^ This means that part of the apparent entropy reduction with respect to the gas phase in ref (76) is probably due to limitation of the free 2D translation; and more than 70% of the internal degrees of freedom of the gas phase is preserved. Alkane chains may be considered a special case since van der Waals bonding to a surface favors molecules lying flat, hindering rotations around the C–C axes. Many other organic molecules are more rigid, and, thus, their internal degrees of freedom are less affected in the adsorbed state. Therefore, for rigid molecules, we consider it a reasonable approximation to assume that the internal degrees of freedom of the gas phase are preserved on the surface, with the exception of the rotation of the molecule as a whole. These gas-phase rotations are included in our program (when using the tabulated data^28^ as well as for other molecules, see eqs 10–13).

This approximation may be somewhat coarse for some molecules of interest such as methanol because these have a torsional mode with a very low rotation barrier, and that mode substantially differs between the gas and the adsorbed molecule. (In the gas phase, the single H of the OH dominates the reduced moment of inertia; in the adsorbed phase, usually the O or OH group will be anchored and the methyl group can rotate; it has a higher moment of inertia than the OH.) Fortunately, the influence of such a “soft” mode on the calculated result may also be still tolerable at least for only one such mode per molecule: comparing the ideal-gas enthalpy and entropy values including all degrees of freedom^48^ of C_2_H_5_OH with eqs 12 and 13, where the soft modes (mainly the rotation around the C–C bond) are ignored, the difference would cause a ≈4% difference of the calculated adsorption energy for a desorption peak at 300 K.

On the other hand, if good estimates for the low-frequency modes of the molecules are available, for example, from DFT calculations, and the sticking coefficient at the desorption temperature can be determined with some accuracy (Section 7.4), our equations pave the way to a high-accuracy determination of the adsorption energy *E*_a_^(0)^ via standard TPD experiments. It should be stressed that *E*_a_^(0)^ is the binding energy, without the vibrational and rotational energy contributions, and, thus, directly comparable with DFT calculations (with zero-point corrections, when required).

### 7.6 Direct Calculation of the Adsorption Energy—How Good Is It?

Assuming a single adsorption energy and given a reasonable estimate for the sticking coefficient *s*_0_ in the low-coverage limit (and the contributions of the vibrational degrees of freedom, when required), our eqs 35–37 directly yield the adsorption energy. To our knowledge, so far, no closed solution to derive the adsorption energy from eq 1 was presented, only an approximation;^4^ otherwise, one had to resort to numerical simulations. Although the width of the desorption peak appears in our equations, we do not use it to determine the pre-exponential factor as in ref (7), we only use it to get the absolute value of the desorption rate at the peak. Thus, the peak width in our equations has a weak influence on the result (the pre-exponential factor scales with the peak width).

These equations are based on the assumption of Langmuir-like sticking, but also useful beyond that restriction, as in the case of a precursor: for the case of CO and a peak at 300 K, the difference in adsorption energy for a first-order (Langmuir) model with *s*_0_ = 1 and coverage-independent *s* = 1 (precursor) is about 2%. Nevertheless, if we apply eq 35 to a TPD simulation for a precursor with constant *s* = 1, we get the adsorption energy correct within 0.7% error in spite of the fact that the equation was derived for Langmuirian sticking. This means that the equations compensate for much of the error that one would get applying the wrong model. This is due to the Δ*T*_fwhm_ term; with a precursor, the peak is wider. If the true sticking coefficient is 0.1, but we assume it as 1 (wrong by an order of magnitude), the error is about 6%, the same as when overestimating the prefactor in a traditional TPD analysis by a factor of 10. By comparison, the naive assumption of a prefactor of 10^13^ s^–1^ would result in underestimating the adsorption energy by a quarter. (This calculation assumes β = 1 K/s, adsorption sites with a 4 Å square lattice, and no vibrations of the adsorbed molecule; the error would be less, but still substantial, when taking realistic vibration frequencies of the adsorbate into account.)

In contrast to other methods that depend on the peak shape and/or width, our eqs 35–37 are also robust toward a distribution of adsorption energies, as long as it only broadens the peak and does not introduce a complex peak structure. Assuming a Gaussian distribution of adsorption energies centered at 109 kJ/mol and a standard deviation of 15 kJ/mol for CO (*s*_0_ = 1; again θ_0_ = 1, β = 1 K/s and *n*_a_ = 6.25 × 10^18^ m^–2^, no adsorbate vibrations) results in a TPD peak at ≈300 K with a FWHM of 84 K, much wider than a 300 K peak for a single CO adsorption energy (105 kJ/mol, FWHM = 15.8 K). Equations 35 or 36 yield the correct (mean) desorption energy within a fraction of a percent for both cases. In a conventional TPD analysis, if TPD traces for multiple coverages are not available, such a wide peak could be interpreted as caused by a very low pre-exponential factor, resulting in a huge error of the adsorption energy. The results of eqs 35–37 are also rather insensitive to an incorrect assumption of whether the molecule dissociates or not; for the case of O_2_ or H_2_, an incorrect assumption would cause an error of ≈1% in the calculated adsorption energy per molecule.

Given their robustness, one may consider eqs 35–37 a kind of “magic formula” for TPD analysis, especially for cases where a variation of coverages is not feasible. Nevertheless, it is advisable to simulate the full TPD curve with the value of *E*_a_^(0)^ obtained and compare it with the experiment (ideally, also for different initial coverages) to check whether the assumptions made are consistent with the experimental data.

### 7.7 Distributions of the Adsorption Energy

In the case of a distribution of adsorption energies (see Section 5), TPD cannot be described by simple first- or second-order kinetics. Recall that the θ or θ^ 2^ factor in the Polanyi–Wigner equation, characteristic for first- or second-order desorption, comes from the entropy derivative for the lattice gas, eq 25. In the general case of a distribution of adsorption energies, the entropy is lower than in eq 25, which describes a lattice gas with all adsorption energies having the same value. As an example, let us assume an energy region where ρ(ε) is constant over a range much wider than *k*_B_*T*. In this case, the configurational entropy of the lattice gas (the entropy of the Fermi gas) is almost independent of ε (or the coverage θ), hence, d*S*_config_/dθ ≈ 0. Then, according to eq 4, *S*_config_ does not contribute to the chemical potential of the adsorbate. This implies that, apart from any coverage dependence of the sticking coefficient *s*, there is no factor θ in the expression for the desorption rate (see eqs 42 and 43). Such a factor would be characteristic for first-order desorption. Of course, we do not obtain a peak shape typical for zero order; according to eq 50, one will instead observe a rather flat region of the desorption rate (μ is a roughly linear function of the temperature, mainly due to the temperature dependence of μ_g0_ in eq 47). This also means that the common assumption of first-order desorption in the sense of the Polanyi–Wigner equation (eq 1) is not justified in the case of a distribution of adsorption energies, and the TPD spectrum is *not* a superposition of first-order spectra for the range of adsorption energies.

**Figure 6:**
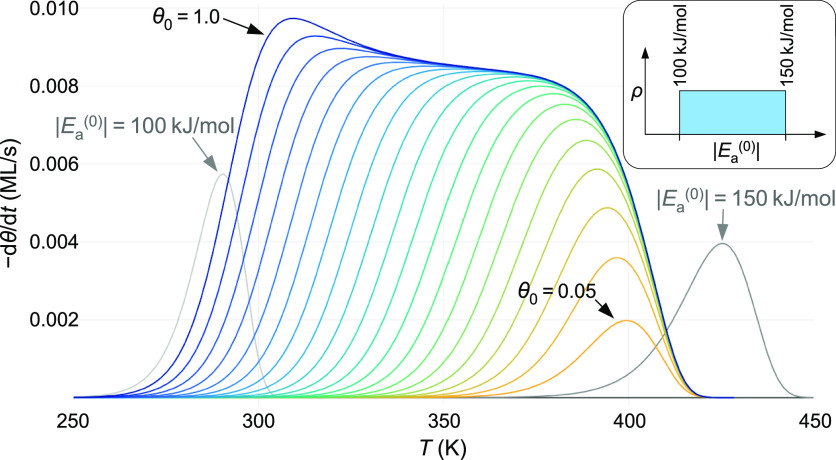
Calculated TPD spectra for a top-hat distribution of adsorption energies *E*_a_^(0)^. The inset shows the distribution. The gas is CO, and the parameters are the same as in Figures 3 and 5, but here assuming Langmuirian sticking with *s*_0_ = 1, which would result in first-order kinetics for a single adsorption energy. The gray curves are for θ_0_ = 0.1 and single adsorption energies of 100 and 150 kJ/mol. (Since these are first-order curves, θ_0_ does not influence the peak position.)

This is exemplified in Figure 6, which shows simulated TPD traces assuming adsorption sites with a flat (top-hat) distribution of energies |*E*_a_^(0)^| between 100 and 150 kJ/mol (inset). Except for the assumption of Langmuirian sticking (*s*_0_ = 1), all other parameters are as shown in Figure 5. The low-temperature side of the TPD spectrum is essentially as expected when assuming a single adsorption energy of 100 kJ/mol. The high-temperature side ends earlier than a TPD spectrum for a single |*E*_a_^(0)^| = 150 kJ/mol, however. This is caused by differences in the configurational entropy of the adsorbates: at low coverage, assuming a single adsorption energy for all sites, the adsorbate molecules have plenty of sites to choose from, and, thus, a high configurational entropy. On the other hand, in the case of a wide distribution of adsorption sites, most sites will be far too unfavorable for binding, and only a small fraction of all adsorption sites have the chance of being taken, leading to a much lower entropy. Then, the adsorbed state is less favorable and the molecules desorb earlier. In the current case, this would be equivalent to increasing the pre-exponential factor by more than an order of magnitude. While the ρ(*E*_a_^(0)^) distribution in Figure 6 is purely hypothetical, the same will also happen for real cases of heterogeneous surfaces: if there is a low concentration of sites with strong binding (such as defects), in a TPD analysis based on the Polanyi–Wigner equation, one would need to apply a very high pre-exponential factor for these sites: the density *n*_a_ of adsorption sites enters the pre-exponential factor in the denominator, see eq 30.

Of course, our model and all these considerations are valid only if the adsorbed phase is in equilibrium, which requires that diffusion barriers are lower than the desorption barrier by many *k*_B_*T*. This is usually the case. Exceptions may occur, for example, if long-range diffusion between different domains would be required to reach equilibrium.

In the present work, we analyze the case of an inhomogeneous lattice gas without interaction, where the adsorption energies are site dependent. This is not the same as a lattice gas with repulsive interactions but with equivalent adsorption sites (the model of refs (18) and (19)). Both cases are somewhat similar because adsorption becomes weaker as the coverage increases. Nevertheless, especially toward low coverage, there are clear differences in the configurational entropy of the adsorbed phase. As an example, let us consider a square lattice with strong, repulsive nearest-neighbor interactions but negligible interactions over larger distances. This leads to weaker adsorption for coverages above 0.5 ML, and so our model will handle this as half of the sites having substantially weaker adsorption. For low coverages, below 0.5 ML, both models, the one with interactions and our assumption of site-dependent energies, will get an almost constant value for the average adsorption energy (that without interactions). The entropy of the two models will differ in the low-coverage limit: in a model with interactions, essentially all sites are available (the excluded area around each adsorbate, i.e., the nearest-neighbor sites of the adsorbates, will add up to only a small fraction of all sites). In a model with half of the sites having a substantially less-favorable energy, half of the sites will be unavailable at low coverage (too high in energy). Thus, in the low-coverage limit, the configurational entropy of the wrong model, with site-dependent energy instead of repulsion, will be lower by *k*_B_ ln 2. For a hexagonal close-packed lattice with strong nearest-neighbor repulsion, the critical coverage where unfavorable adsorption configurations comes into play is 1/3 ML;^77^ thus, the entropy error of using a model based on site-dependent adsorption would approach *k*_B_ ln 3 at low coverages. Fortunately, the concomitant error in adsorption energies will be rather small (|*E*_a_^(0)^| overestimated by about 2 and 3% in these two cases; the same error as caused by a factor of 2 or 3 in the prefactor of a standard TPD analysis). Therefore, unless extremely high accuracy is needed, at least for short-range repulsive interactions, the simple model of site-dependent adsorption energies is also a useful approximation for the case of interacting adsorbates. In contrast to the method of Kreuzer et al.,^17−20^ our model of site-dependent adsorption energies requires no modeling of interactions between adsorbates, so we consider it easier to use, especially if the details of the atomic-scale structure are unknown. Most other methods of analyzing TPD data, like the inversion analysis,^6^ do not distinguish between repulsion and a distribution of adsorption energies as the underlying models are more coarse than ours. Especially for adsorption at surfaces with a rather low corrugation of the potential energy surface, the “stiffening” of the hindered translation modes with increasing coverage due to repulsive interactions between the adsorbates may have more impact on the final result than the incorrect description of the configurational entropy by assuming site-dependent adsorption energies instead of repulsive interactions.

An example of applying our method (and program) to experimental (not simulated) data is provided in the Supporting Information. It discusses the case of CO desorption from Fe_3_O_4_(001), where CO–CO repulsion leads to weakening of adsorption above 0.5 ML coverage.^78^

While a distribution of adsorption energies can approximate repulsive interactions, this is not true for attractive interactions, which need to be modeled as such (as done in refs (17)–^20^). The results of our computer program can indicate the presence of attractive interactions, however: if a TPD peak is sharper than the simulation for a sharp adsorption energy, and this cannot be explained by wrong assumptions (an incorrect assumption of constant sticking or too high a vibrational entropy for the adsorbate), attraction between the adsorbate molecules is a likely reason. Attractive interactions should be considered especially at rather low temperatures. (The energy scale of attractive interactions is given by the multilayer peak; thus, one should consider attractive interactions up to roughly 2 times the temperature of the multilayer peak.) Also, a peak shift to higher temperature with increasing coverage (as one would get with a desorption order below unity), or no shift with increasing coverage in cases where our program predicts a downshift, could be considered an indication of attractive interactions.

## 8 Conclusions

We have presented equations for the analysis of TPD data, following the approach of Payne and Kreuzer et al.,^17−24^ with simplifications (no interactions) and also extending this previous work. We include the case of well-defined adsorption sites with a distribution of adsorption energies, but no interaction between adsorbates. We argue that our model can also be used as an approximation for short-range repulsive interactions between adsorbates in case the atomic-scale details required for handling the interactions by the model of Payne and Kreuzer are unknown. In simple cases, eqs 35–37 directly yield the adsorption energy from the peak temperature, peak width, sticking probability, and thermodynamic data of the adsorbate. For more complex cases, our paper is accompanied by a computer program for simulating TPD spectra and extraction of adsorption energies. The program can also determine the adsorption energy distribution from a single TPD spectrum at saturation coverage.

For the case of a lattice gas with a single adsorption energy, we have described the conditions for validity of the Polanyi–Wigner equation with a coverage-independent pre-exponential factor. If the assumption of first-order kinetics is justified, except for hydrogen, typical pre-exponential factors for small molecules are in the order of 10^15^ to 10^17^ s^–1^, which is higher than often expected. For a distribution of adsorption energies, even if the sticking coefficient would lead to first-order kinetics for a single adsorption energy, the TPD spectra clearly differ from a superposition of first-order TPD spectra for the adsorption energies in the distribution. We have also shown that some TPD analysis methods based on the Polanyi–Wigner equation (peak-shape-based methods, leading edge analysis) can lead to substantial errors of the adsorption energy if the sticking does not follow Langmuir adsorption kinetics. Such analysis methods may be partly responsible for reports of an apparent compensation effect between the pre-exponential factor and adsorption energies in the literature. We have argued that the analysis methods described in the present work can lead to high-accuracy determinations of adsorption energies if the sticking probability at the desorption temperature and the vibration frequencies of the adsorbate are known.

## Supporting Information Available

Application example: TPD analysis for CO/Fe_3_O_4_(001) (PDF)
Computer program (HTML with JavaScript) for simulation and analysis of TPD spectra assuming a lattice gas (ZIP)

## Formula symbols

*A*: surface area
*a*: excluded area per adsorbed molecule (ideal 2D gas)
*d*: distance between energy minima at the surface
*E*_a_^(0)^: adsorption energy (without *E*_a,int_)
*E*_a,int_: rotational & vibrational energy per adsorbate
*E*_a,trans_: translational energy per adsorbate (ideal 2D gas)
*E*_b_: energy barrier for diffusion
*E*_g,int_: rotational & vibr. energy per gas-phase molecule
*E*_g,rot_: energy of rotation per gas-phase molecule
*E*_vib_: energy of a vibrational degree of freedom
Δ*E*: energy difference or desorption barrier
Δ*E*_ad_: barrier for activated adsorption
Δ*E*_int_: rot. & vibr. energy difference, gas minus adsorbate (per molecule)
*f*_corr_: correction factor (Richardson–Lucy)
*G*: Gibbs free energy
*h*: Planck’s constant
*H*: enthalpy
*H*_a_: enthalpy per adsorbate
*H*_g_: enthalpy per gas-phase molecule
*I*: moment of inertia or intensity
*k*_B_: Boltzmann’s constant
*k*′: convolution kernel
*m*: mass of (gas-phase) molecule
*n*: desorption order
*n*_a_: density of adsorption sites (per area)
*N*: number of molecules
*N*_d_: number of equivalent fragments when dissociating, otherwise 1
*p*: (gas) pressure
*p*_0_: reference pressure (1 bar)
*s*: sticking coefficient
*s*_0_: initial sticking coefficient (at θ → 0)
*S*: entropy
*S*_a_: entropy per adsorbate
*S*_g0_: entropy per gas-phase molecule at *p*_0_
*S*_a,int_: rotational & vibrational entropy per adsorbate
*S*_a,trans_: entropy of translation per adsorbate (ideal 2D gas)
*S*_conf_: configurational entropy of adsorbed phase
*S*_g,int_: rotational & vibr. entropy per gas-phase molecule
*S*_g,rot_: entropy of rotation per gas-phase molecule
*S*_vib_: entropy of a vibrational degree of freedom
Δ*S*_int_: rot. & vibr. entropy difference, gas minus adsorbate (per molecule)
*t*: time
*T*: temperature
Δ*T*_fwhm_: full width at half-maximum of a peak
*V*: volume
β: heating rate d*T*/d*t*
ε: adsorbate energy (Fermi–Dirac statistics)
θ: coverage (molecules per adsorption site)
θ_0_: initial coverage
Λ: thermal de Broglie wavelength
μ: chemical potential
μ_a_: chemical potential per adsorbate
μ_g_: chemical potential per gas-phase molecule
μ_g0_: chemical potential at *p*_0_ (gas phase)
ν: vibration frequency
ν_0_: pre-exponential factor (rate constant)
ρ: adsorption energy distribution
σ: number of indistinguishable rotational orientations
