# Supplementary material for: Analysis of Temperature-Programmed Desorption via Equilibrium Thermodynamics
Source: ACS Phys Chem Au. 2022 Nov 15;3(1):44–62. doi: 10.1021/acsphyschemau.2c00031 (PMC9881163; doi:10.1021/acsphyschemau.2c00031)
Supplement: Supplementary file 1 — pg2c00031_si_001.pdf [file pg2c00031_si_001.pdf]

SUPPLEMENTARY MATERIAL

# Analysis of Temperature-Programmed Desorption via Equilibrium Thermodynamics

ACS Physical Chemistry Au (2022)
**DOI:** 10.1021/acsphyschemau.2c00031

Michael Schmid,* Gareth S. Parkinson, and Ulrike Diebold

*Institute of Applied Physics, TU Wien, 1040 Vienna, Austria*

## A Case study: CO on $Fe_3O_4$(001)

As an example of applying our method, let us consider the case of CO desorption from $Fe_3O_4$(001).[1] The surface consists of rows of octahedral $Fe^{3+}$ atoms with an Fe–Fe distance of $d = 297$ pm and rather wide spacing between the rows (594 pm), see the bottom inset in Figure S1.[2] Each Fe atom in the rows is a potential adsorption site for CO, resulting in $n_a = 5.7 \times 10^{18}$ m$^{-2}$ corresponding to 1 ML. (The tetrahedral Fe atoms between these rows are both coordinatively saturated and recessed, thus not accessible for CO.) The TPD spectra (Figure S1) indicate that the CO molecules tend to avoid nearest-neighbor (NN) sites up to a coverage of 0.5 ML (the dip at 75 K), then adsorption gets weaker due to a strong CO-CO repulsion on NN sites. Small peaks above 120 K are due to defects.[1] Analysis of these data using the inversion method[3] resulted in two very different pre-exponential factors, $10^{13}$ and $10^{18}$ s$^{-1}$, for coverages below and above 0.5 ML, respectively. The increase of the prefactor at 0.5 ML leads to the astonishing result of a non-monotonous behavior of the desorption barrier (decreasing from $\approx 0.27$ to 0.20 eV from 0 to 0.5 ML, then again from $\approx 0.27$ to 0.23 eV in the 0.5–1 ML range). This result was rationalized by the fact that the (Gibbs) free energy, not $E_a^{(0)}$, should decrease monotonously during a TPD ramp [see eq (4) of the main text; also $\mu$ decreases], and the adsorbate was assumed to be a 1D gas with a high entropy at coverages below 0.5 ML.[1] In hindsight, the 0.07 eV jump of the barrier at 0.5 ML coverage is hard to rationalize as a mere entropy effect, however. It corresponds to about 11 $k_B T$ or a factor of $\approx 5 \times 10^4$ in the number of microstates according to Boltzmann's entropy formula. This is several orders of magnitude too high for the transition from a 1D ideal gas to a 1D lattice gas ($\Lambda = 38$ pm at 75 K; the difference between our lattice gas model without vibrations and the 1D Sackur–Tetrode equation for an ideal 1D gas corresponds to about 1½ orders of magnitude in the prefactor or the number of microstates).

The upper inset in Figure S1 shows the $\rho(E_a^{(0)})$ distribution obtained by analyzing the CO/$Fe_3O_4$(001) data with our program, using the 1 ML TPD curve as input. The calculated TPD traces for this distribution are shown on top of the (red) experimental curves. It can be seen that the experimental data can be reasonably well reproduced without the assumption of any soft vibration

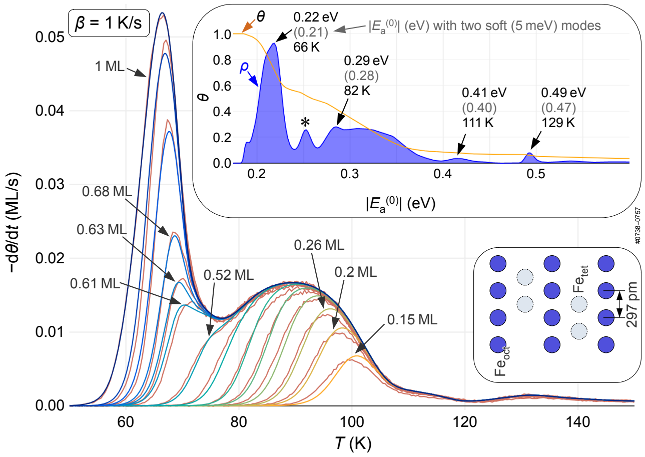


Figure S1: TPD of $CO/Fe_3O_4(001)$. The red curves are experimental TPD data up to 1 ML from Ref. [1], and the other colors (orange to dark blue) are simulated data using the adsorption energy distribution (upper inset) calculated from the 1 ML curve, assuming Langmuirian sticking ($s_0 = 1$) and no influence of vibrations. Coverages of selected curves correspond to the calculated curves and slightly differ from those in Ref. [1] due to the use of ionization probability correction (see section 6.1 of the main paper) and a different background subtraction method. The upper inset shows the calculated adsorption energy distribution as well as the integral over $\rho(E_a^{(0)})$ (the cumulative distribution function, orange). The labels mark selected $|E_a^{(0)}|$ values together with the corresponding desorption temperatures according to eq (47) of the main text. The peak marked with an asterisk is possibly an artifact, see text. The bottom inset is a schematic top view of the surface structure (O atoms are omitted). The $Fe_{oct}$ atoms are potential adsorption sites for CO.

modes or additional entropy. Small deviations of the low-coverage curves at 90–105 K are partly due to slight variations of the background with coverage. (Note that the experimental 0.15 ML curve does not reach the hull of the others between 105 and 120 K; this would not occur in a TPD spectrum as long as all adsorbates are in equilibrium.) Nevertheless we should examine the sensitivity of our analysis towards increased entropy of the adsorbate at low coverage. Assuming two very soft hindered-translation modes (5 meV or 40 $cm^{-1}$ each) has no appreciable impact on the simulated spectra and only a very mild influence on the calculated values of the adsorption energies (values in brackets for selected peaks in the upper inset of Figure S1).

The calculated adsorption energies in the high-coverage regime (0.19–0.24 eV assuming negligible contributions of the vibrations) are comparable with those of the previous inversion analysis[3] of the same data (0.23–0.27 eV).[1] We find substantially stronger bonding at lower coverages (0.26–0.38 eV vs. 0.20–0.27 eV in the previous work). Since our analysis is based on the chemical potential, which decreases monotonously during a TPD ramp, the non-monotonicity of the previous analysis[1] (jump of $E_a^{(0)}$ at half-monolayer coverage) is gone.

The high-coverage peak in the $\rho(E_a^{(0)})$ distribution has a finite width, which is not a result of the finite energy resolution of the method. (In our program, one could easily verify this in "expert mode" by more iterations and less noise suppression of the Richardson-Lucy refinement.) The peak width may be caused by CO molecules lowering their energy by bending away from each other as long as the rows of adsorption sites are not fully occupied. Alternatively, the assumption of Langmuirian sticking may be incorrect.[†] The defect peaks above 0.4 eV in the $\rho(E_a^{(0)})$ distribution are rather sharp, indicating well-defined defect sites.

It is less clear why there is a rather wide distribution of adsorption energies below 0.5 ML (between 0.26 and 0.38 eV), and not a single adsorption energy on the Fe rows in the low-coverage regime. This distribution was originally explained as CO–CO repulsion over rather long distances.[1] The physics behind such a long-range repulsion is unclear, however. We consider it likely that the distribution of adsorption energies between 0.26 and 0.38 eV is not only due to repulsion but also due to the influence of defects. Domain boundaries,[5] interstitial Fe atoms[6] and adsorbed hydro-

[†] A precursor model with constant sticking, $s = 1$, does not nicely fit the low-temperature onset of the 1 ML curve; a Kisliuk precursor model[4] with $s_0 = 0.9$ [equation in the "Formula: $s(\theta, T)$" field of Figure 3 of the main paper] results in a fit as good as Langmuirian sticking; the only difference to the Langmuirian model is a narrower $\rho(E_a^{(0)})$ peak at 0.22 eV. Assuming this sticking model shifts the peaks in the adsorption energy distribution by less than 0.01 eV.

gen[7] all lead to $Fe^{2+}$ sites; CO adsorption on these sites is expected to be stronger than on $Fe^{3+}$. In addition, one may also consider an influence of $Fe^{2+}$ defects on nearby $Fe^{3+}$ sites. We should note that the typical $Fe^{2+}$ concentration is substantially higher than the 1.65% of the $\approx$ 130 K peak; thus it is unlikely that only the peaks at 130 K and higher are affected by $Fe^{2+}$.

Our analysis provides no direct answer to the question whether the CO molecules form a lattice gas or a 1D gas with free translation along the Fe rows at low coverages ($\theta < 0.5$). The entropy difference between these two models is about $4.3k_B$ (it can be added as "extra entropy" in our program); this causes no substantial change concerning the agreement between simulated and measured data, it only leads to a downshift of the $\rho(E_a^{(0)})$ curve by 0.03 eV (the width of the energy distribution mentioned above remains the same). Indirect evidence speaks for a lattice gas, however: When assuming a low energetic cost to reach a CO position between two Fe atoms, as required for an (almost) ideal 1D adatom gas, there would not be such a clear change of $|E_a^{(0)}|$ (and a dip in the TPD spectra) around 0.5 ML. A coverage somewhat above 0.5 ML could be easily attained by evenly distributing the CO molecules along the rows (a Frenkel-Kontorova model with strong repulsion and weak corrugation of the potential energy). This would avoid the strong repulsion between CO molecules at NN Fe sites required in a lattice-gas model at $\theta > 0.5$ ML, and, therefore, lead to a more gradual weakening of the adsorption above 0.5 ML.

As a side note, the upper inset of Figure S1 shows a small peak in $\rho(E_a^{(0)})$ at $\approx$ 0.25 eV, at $\theta =$ 0.52 ML and marked with an asterisk. This peak encompasses a coverage range of about 0.06 ML. This peak corresponds to a temperature slightly below the dip at $\approx$ 75 K in the spectrum. The peak would disappear when reducing the experimental peak intensities around 73 K by 20%, i.e., assuming that the experiment has added a tail to the 66 K peak due to finite pumping speed. While such tails can occur at higher ramp rates $\beta$, the agreement between the TPD curves with different coverages (down to 0.68 ML) around 73 K is good, allowing us to exclude that the 0.25 eV peak in $\rho(E_a^{(0)})$ is entirely an experimental artifact. If the coverage determination is slightly inaccurate, and this $\rho(E_a^{(0)})$ peak is entirely above 0.5 ML, it may correspond to locally full coverage at defect sites, where CO is bound more strongly than at regular sites. We consider it also possible that the 0.25 eV peak is an artifact of using the wrong model; we have to bear in mind that our analysis does not capture the exact physics. (It is based on adsorption-site dependent energies, not on repulsive interaction. Strictly speaking, this is not a mathematically correct description). Nevertheless, we consider the present analysis clearly more trustworthy than methods based on the assumption of a superposition of first-order spectra, which lead to a substantial non-monotonicity of the desorption energies at 0.5 ML.

## REFERENCES


(1) Hulva, J.; Jakub, Z.; Novotny, Z.; Johansson, N.; Knudsen, J.; Schnadt, J.; Schmid, M.; Diebold, U.; Parkinson, G. S. Adsorption of CO on the $Fe_3O_4$(001) surface. *J. Phys. Chem. B* **2018**, *122*, 721–729, DOI: 10.1021/acs.jpcb.7b06349.

(2) Bliem, R.; McDermott, E.; Ferstl, P.; Setvin, M.; Gamba, O.; Pavelec, J.; Schneider, M. A.; Schmid, M.; Diebold, U.; Blaha, P.; Hammer, L.; Parkinson, G. S. Subsurface cation vacancy stabilization of the magnetite (001) surface. *Science* **2014**, *346*, 1215–1218, DOI: 10.1126/science.1260556.

(3) Tait, S. L.; Dohnálek, Z.; Campbell, C. T.; Kay, B. D. n-alkanes on MgO(100). I. Coverage-dependent desorption kinetics of n-butane. *J. Chem. Phys.* **2005**, *122*, 164707, DOI: 10.1063/1.1883629.

(4) Kisliuk, P. The sticking probabilities of gases chemisorbed on the surfaces of solids. *J. Phys. Chem. Sol.* **1957**, *3*, 95–101, DOI: 10.1016/0022-3697(57)90054-9.

(5) Parkinson, G. S.; Manz, T. A.; Novotný, Z.; Sprunger, P. T.; Kurtz, R. L.; Schmid, M.; Sholl, D. S.; Diebold, U. Antiphase domain boundaries at the $Fe_3O_4$(001) surface. *Phys. Rev. B* **2012**, *85*, 195450, DOI: 10.1103/PhysRevB.85.195450.

(6) Gamba, O.; Hulva, J.; Pavelec, J.; Bliem, R.; Schmid, M.; Diebold, U.; Parkinson, G. S. The role of surface defects in the adsorption of methanol on $Fe_3O_4$(001). *Top. Catal.* **2017**, *60*, 420–430, DOI: 10.1007/s11244-016-0713-9.

(7) Parkinson, G. S.; Novotný, Z.; Jacobson, P.; Schmid, M.; Diebold, U. Room temperature water splitting at the surface of magnetite. *J. Am. Chem. Soc.* **2011**, *133*, 12650–12655, DOI: 10.1021/ja203432e.
