# Supplementary material for: Analysis of Temperature-Programmed Desorption via Equilibrium Thermodynamics
Source: ACS Phys Chem Au. 2022 Nov 15;3(1):44–62. doi: 10.1021/acsphyschemau.2c00031 (PMC9881163; doi:10.1021/acsphyschemau.2c00031)
Supplement: Supplementary file 2 — pg2c00031_si_002.zip [file pg2c00031_si_002.zip › TPDanalysis_v10.html]

TPD Analysis for an Ideal Lattice Gas


# TPD Analysis for a Lattice Gas

ERROR: You have to enable JavaScript in your browser.


|  |  |  |  |
| --- | --- | --- | --- |
| Calculation for what | E\_ads given T\_peak given Distribution of E\_ads given TPD curve given, get E\_ads distrib. | | Wait (calculating)... |
| Units | J/mol eV |
| Gas |  | a)   dissociates | |
| Atom types, *x*, *y*, *z* | C 0 0 0 O 0 0 1.13 | | units  m |
| Ramp rate *β* |  | K/s |
| Adsorption sites/area *n*a |  | m⁻² cm⁻² nm⁻² |
| *T*peak |  | K; FWHM =  K | |
| |*E*ads0| |  | J/mol   b) |
| |*E*ads0|, fraction | 1e5,1 | | J/mol   b) |
| Adsorbate vibrations *E*vib |  | | cm-1   c) |
| Extra entropy *S*ad,extra/*R* |  | | d) |
| Initial coverage *θ*0 |  |
| Multiple coverages |  | | e) |
| Sticking:    Constant: *s* |  | (mobile precursor) | |
| Per free site: *s*0 |  | (Langmuir-type adsorption) | |
| Formula: *s*(*θ*, *T*) |  | | f) |

Plot experimental
Experimental TPD curve(s)
for comparison: *T* (K), rate, [*T*, ] rate...  

Ionization probability correction (√*T*)
g)  
230,0.01
232,0.04
234,0.08
236,0.16
238,0.32
240,0.50
242,0.62
244,0.76
246,0.93
248,1.14
250,1.38
252,1.68
254,2.03
256,2.46
258,2.95
260,3.54
262,4.24
264,5.05
266,6.00
268,7.11
270,8.41
272,9.90
274,11.63
276,13.61
278,15.88
280,18.46
282,21.39
284,24.69
286,28.40
288,32.52
290,37.08
292,42.08
294,47.50
296,53.32
298,59.48
300,65.87
302,72.38
304,78.81
306,84.91
308,90.38
310,94.82
312,97.80
314,98.81
316,97.33
318,92.92
320,85.27
322,74.41
324,60.83
326,45.67
328,30.65
330,17.74
332,8.46
334,3.15
336,0.85
338,0.15
340,0.02

Expert mode:
Number of sharpening iterations 
Noise suppression (0–10) 
 h)  
Output: |*E*ads0| (J/mol), fraction, *θ*exp,
*ρ*(|*E*ads0|), *ρ*raw(|*E*ads0|),
*θ*, *T*  
 

a)
For the predefined gases except H2, data are inaccurate below 100 K. For lower temperatures, better select 'OTHER' and enter the molecular structure.
Dissociation is only supported for molecules dissociating into two atoms of equal type (H2, D2, N2, and O2).  
b) *E*ads0 is the adsorption energy for *T* = 0, i.e., without vibrational energies of the adsorbate. Energy per atom when dissociating.
Each line should contain two numbers, |*E*ads0|, *f*, where *f* is the fraction or relative number of adsorption sites with this adsorption energy.
(You need not care about normalization; the program will normalize it such that the sum of all *f* values becomes unity.) |*E*ads0| values must be given in ascending order.
Further data columns will be ignored.  
c) Comma-separated list, leave empty if none.
Enter all modes for the adsorbate if the gas is one of the pre-defined ones.
For 'OTHER' gases, omit vibration modes that are equal for the gas phase and the adsorbed molecule.
Enter vibration modes present only in the adsorbed molecules as positive numbers and modes present only in the gas phase as negative numbers.
Modes with high *E*vib = *hν* (above a few *k*B*T*) can be ignored.  
d) Adsorbate entropy not related to site occupation and vibrations, divided by *R*.
E.g., ln(3) for three distinguishable azimuthal orientations. See f) for how to enter a formula.  
e) Enter the step Δ*θ*0, or a list of initial coverages *θ*0(1), *θ*0(2),... (best in descending sequence).
These coverages should be less than the *θ*0 value of the main calculation.  
f) Variables: θ or th, T, and s (output; optional if a simple expression is given).
Declare own variables with var. Predefined constants h (Planck), k (Boltzmann), R (gas), and e (elementary charge, eV/J).
Functions: sqrt, sqr, ln or log, exp, pow(base, exponent), and JavaScript
Math functions.
Examples: sqr(1-θ) or var x=10\*(1-th); s=x/(1+x).
  
g) Assumes that the ionization probability in the mass spectrometer is inversely proportional to the velocity, which is proportional to √*T*.
This correction should be applied only if the desorbing molecules directly enter the mass spectrometer, without being reflected at any surfaces.
The correcion is also inaccurate in case of a barrier on adsorption, causing temperature-dependent sticking.
  
h) Select 'Expert mode' to control the fitting process used for determination of the energy distribution (Richardson–Lucy method).
 The raw energy distribution
is iteratively refined to better fit the experimental data. A high *number of iterations* (default 50) can lead to a better fit,
but takes more computing time and may also result in isolated desorption energies even in case of a continuous distribution of energies, to better fit the experimental noise.
If you get at least one rather sharp peak in the energy distribution and the calculated TPD peak corresponding to it is not as sharp as the expermental one, consider increasing the number of iterations.
The *noise suppression* (default value 5) reduces sharpening broad distributions of the adsorption energy.  
In expert mode, the energy distribution plot conains additional information:
The raw correction factor is the ratio of experimental and calculated peak intensity in the last interation of the Richardson–Lucy refinement,
and the correction factor actually applied is obtained by smoothing this raw factor.
The plot also shows the raw energy distribution *ρ*raw and an intermediate result for
*ρ*(|*E*ads0|), to visualize how it evolves from *ρ*raw to the final result.
 Hide footnotes

Legend
off
left
right
outside

Full height  

Export plot: *T* =
– K,   
 ×
 pxl,  Line width
 pxl   

  
*T*, Calculated desorption rate(s)  
 

Author: Michael Schmid, IAP/TU Wien.  
This work is licensed under
GNU General Public License (GPLv3) and
Creative Commons Attribution-ShareAlike 4.0 (CC-BY-SA 4.0).
The plot library *Plotly* is licensed under the MIT license.
  
When using and/or modifying this program for scientific work please cite our paper:  
Michael Schmid, Gareth S. Parkinson, and Ulrike Diebold, *Analysis of temperature-programmed desorption via equilibrium thermodynamics*,  
ACS Phys. Chem. Au (2022), doi: 10.1021/acsphyschemau.2c00031
